# miR126-5p Downregulation Facilitates Axon Degeneration and NMJ Disruption via a Non–Cell-Autonomous Mechanism in ALS

**DOI:** 10.1523/JNEUROSCI.3037-17.2018

**Published:** 2018-06-13

**Authors:** Roy Maimon, Ariel Ionescu, Avichai Bonnie, Sahar Sweetat, Shane Wald-Altman, Shani Inbar, Tal Gradus, Davide Trotti, Miguel Weil, Oded Behar, Eran Perlson

**Affiliations:** ^1^Department of Physiology and Pharmacology, Sackler Faculty of Medicine, Tel Aviv University, Tel Aviv 69978, Israel,; ^2^Department of Developmental Biology and Cancer Research, Hebrew University of Jerusalem, Jerusalem 9190401 Israel,; ^3^Department of Cell Research and Immunology, Tel Aviv University, Tel Aviv 69978, Israel, and; ^4^Jefferson Weinberg ALS Center, Vickie and Jack Farber Institute for Neuroscience, Department of Neuroscience, Thomas Jefferson University, Philadelphia, Pennsylvania 19107

**Keywords:** ALS, axon degeneration, microfluidic chambers, miRNA, NMJ, Sema3A

## Abstract

Axon degeneration and disruption of neuromuscular junctions (NMJs) are key events in amyotrophic lateral sclerosis (ALS) pathology. Although the disease's etiology is not fully understood, it is thought to involve a non–cell-autonomous mechanism and alterations in RNA metabolism. Here, we identified reduced levels of miR126-5p in presymptomatic ALS male mice models, and an increase in its targets: axon destabilizing Type 3 Semaphorins and their coreceptor Neuropilins. Using compartmentalized *in vitro* cocultures, we demonstrated that myocytes expressing diverse ALS-causing mutations promote axon degeneration and NMJ dysfunction, which were inhibited by applying Neuropilin1 blocking antibody. Finally, overexpressing miR126-5p is sufficient to transiently rescue axon degeneration and NMJ disruption both *in vitro* and *in vivo*. Thus, we demonstrate a novel mechanism underlying ALS pathology, in which alterations in miR126-5p facilitate a non–cell-autonomous mechanism of motor neuron degeneration in ALS.

**SIGNIFICANCE STATEMENT** Despite some progress, currently no effective treatment is available for amyotrophic lateral sclerosis (ALS). We suggest a novel regulatory role for miR126-5p in ALS and demonstrate, for the first time, a mechanism by which alterations in miR126-5p contribute to axon degeneration and NMJ disruption observed in ALS. We show that miR126-5p is altered in ALS models and that it can modulate Sema3 and NRP protein expression. Furthermore, NRP1 elevations in motor neurons and muscle secretion of Sema3A contribute to axon degeneration and NMJ disruption in ALS. Finally, overexpressing miR126-5p is sufficient to transiently rescue NMJ disruption and axon degeneration both *in vitro* and ***in vivo***.

## Introduction

Amyotrophic lateral sclerosis (ALS) is a lethal neurodegenerative disease that affects motor neurons (MNs) in the cortex, brainstem, and spinal cord (SC) (Mulder et al., 1986; Peters et al., 2015). It is characterized by neuromuscular junction (NMJ) disruption, MN axon degeneration, and neuronal death (Frey et al., 2000; Fischer et al., 2004; Moloney et al., 2014). Despite some progress, currently no effective treatment is available for ALS. The diversity of ALS-related mutations has given rise to the use of numerous animal models with diverse phenotypes, ranging from no effect on MN function to severe progressive paralysis (Philips and Rothstein, 2015). Approximately 20% of familial ALS is accounted for by mutations in the superoxide dismutase 1 (*SOD1*) gene (Scrutton et al., 1992; Rosen et al., 1993; Gurney et al., 1994). Mutations in the *SOD1* gene (mSOD1) have also been described in sporadic cases (sALS) (Rakhit et al., 2004). Other mutations found in ALS patients include hexanucleotide expansion repeats in the *C9orf72* locus, which lead to various dipeptide repeats (e.g., proline-arginine or glycine-arginine repeats: PR_50_ and GR_50_, respectively), and in the gene encoding the TDP43 RNA binding protein (Buratti, 2015; Wen et al., 2017). An efficient method for studying NMJ stability and health *in vitro* is by using the microfluidic chamber (MFC) system, which allows the culture of primary myocytes in one compartment, and MNs in the other, thus setting up conditions conducive to generating active NMJs (Zahavi et al., 2015).

The neurodegeneration that occurs in ALS is considered to be a non–cell-autonomous process involving interactions between the neuron and its diverse extracellular microenvironments via an unknown mechanism (Ilieva et al., 2009; Tsitkanou et al., 2016). Although the molecular basis for neuronal dysfunction and death in ALS is still poorly understood, it may be due to alterations in the nature of the extracellular signaling pathways that switch from prosurvival to toxic (Ilieva et al., 2009; Perlson et al., 2009). Numerous studies support the notion that multiple tissues outside the CNS, including skeletal muscle (Dupuis et al., 2006; Tsitkanou et al., 2016), astrocytes (Nagai et al., 2007), and microglia (Lee et al., 2016), contribute to ALS pathologies. Alterations in RNA metabolism and microRNAs (miRs) can contribute to, and also be part of, mechanisms that initiate the disease (Lemmens et al., 2010; Emde and Hornstein, 2014). miRs are post-transcriptional regulators that play an important role in many cellular processes, such as axon growth and retraction, and were demonstrated to be involved in many diseases, including neurodegenerative diseases, such as ALS (Hawley et al., 2017; Molasy et al., 2017). Alterations in miR expression profile were identified specifically in axons of ALS models (Rotem et al., 2017), as well in muscles leading to increasing attempts to either use or target miRs as therapeutic strategies (Di Pietro et al., 2017). Therefore, it is reasonable to assume that alterations in RNA and miRNA metabolism, of both MNs and neighboring cells, can regulate a secreted destabilizing signal, which in turn, facilitates axon degeneration and NMJ disruption.

Semaphorin3A (Sema3A) was initially identified as a repellent guidance molecule (Luo et al., 1993; Worzfeld and Offermanns, 2014). However, later works showed that it can also induce neuronal cell death of sympathetic, sensory, retinal, and cortical neurons (Nakamura et al., 2000; Shirvan et al., 2002; Ben-Zvi et al., 2008; Jiang et al., 2010). Neuropilin1 (NRP1) has been shown to be the receptor binding component for Sema3A as well as some other Type 3 Semaphorins (Kolodkin et al., 1997). Sema3A was found to be upregulated following CNS injury as well as in several neurodegenerative diseases (Kaneko et al., 2006; Van Battum et al., 2015). Importantly, Sema3A was found to be upregulated in terminal Schwann cells of the *SOD1^G93A^* transgenic mouse model for ALS and in the motor cortex of ALS patients (De Winter et al., 2006; Körner et al., 2016), suggesting that it plays a toxic role in disease pathology and progression.

Here we demonstrated that alterations in miR126-5p result in upregulation of Type 3 Semaphorins and its cobinding receptor NRP1 in muscles and MN axons of ALS models, respectively. We further demonstrate *in vitro* and *in vivo* the contribution of this pathway to axon degeneration and NMJ disruption in ALS models.

## Materials and Methods

### 

#### 

##### Animals and vector injections.

HB9::GFP (stock #005029) mice were originally obtained from The Jackson Laboratory. The colony was maintained by breeding with ICR mice. *SOD1^G93A^* (stock #002726) mice were originally obtained from The Jackson Laboratory and maintained by breeding with C57BL/6J mice. Genotyping was performed using the PCR (KAPA Biosystems); DNA samples were generated from ear or tail tissue biopsies. All injection procedures were performed on presymptomatic ∼P60 mice. Mice were first anesthetized using a mixture of xylazine and ketamine. Next, 100 μl of Neurobasal-containing X10 concentrated lentiviruses (6 × 10^9^ titer units) was injected into the gastrocnemius (GC) muscles using a 1 ml syringe and a 25G needle. A pLL-miR126-5p-GFP construct was injected into the right hind foot, whereas a pLL-miR142-GFP construct was injected into the left hind foot. All animal experimentations were approved by the Tel Aviv University Animal Ethics Committee.

##### MFC preparation.

Polydimethylsilxane MFCs were designed and cast as described previously (Ionescu et al., 2016). After the wells were punched, a small “cave” was created in the explant well near the grooves using a 25G needle, keeping the explant in place. Microfluidic devices were cleaned of surface particles using adhesive tape and were sterilized in 70% ethanol for 15 min. Devices were completely dried under sterile conditions using UV radiation and then attached to a sterile 60 mm plastic dish (Nunc) with gentle pressure, and the margins were sealed with polydimethylsilxane before incubation at 70°C for 30 min to prevent the detachment of the chamber. Muscle channels were coated with Matrigel diluted 1:10 with DMEM containing 2.5% penicillin-streptomycin-nystatin (PSN) for 30 min at 37°C, before filling the muscle wells with 150 μl of Bioamf-2 medium. The explant well and channel were filled with 150 μl of 1.5 ng/ml poly-dl-ornithine (P-8638, Sigma-Aldrich) in PBS overnight and then replaced with 150 μl laminin (L-2020, Sigma-Aldrich), 1:333 in deionized distilled water overnight. One day before plating the SC explant, laminin was replaced with explant medium containing Neurobasal (Invitrogen) supplemented with 2% B27 (Invitrogen), 1% penicillin-streptomycin (Biological Industries), 1% Glutamax (Invitrogen), and 25 ng/ml BDNF (Alomone Labs), until the day on which coculturing began.

##### Fluorescence microscopy and image analysis.

All confocal images were captured using a Nikon Ti microscope equipped with a Yokogawa CSU X-1 spinning disc and an Andor iXon897 EMCCD camera controlled by Andor IQ2 software. Epifluorescence was imaged using the same microscope in bright-field mode, and images were captured with an Andor Neo sCMOS camera, or at a FLoid benchtop imaging station (Invitrogen). TIRF images were captured using a TILL photonics iMIC microscope (FEI Munich) with an Andor iXon897 EMCCD camera. All live-imaging assays were performed in a humidified 5% CO_2_ incubation chamber.

##### Western blotting.

Muscle and sciatic nerve (SN) tissues of both sexes were excised and homogenized in lysis buffer containing PBS, 1% Triton X-100 (Sigma-Aldrich), and 1× protease inhibitors (Roche Diagnostics), followed by centrifugation and collection of the supernatant. Protein concentration was determined using the Bio-Rad Protein Assay. Protein samples were denatured by boiling in SDS sample buffer, which were then electrophoresed in 10% polyacrylamide gels (SDS-PAGE). Proteins were transferred to a nitrocellulose membrane and then immunoblotted with appropriate primary antibodies: Sema3A (Abcam, ab23393; 1:1000); NRP1 (Abcam, ab81321; 1:1000); Sema3B (Abcam, ab48197; 1:2000); NRP2 (Cell Signaling D39A5, 1: 1000); GFP (Abcam ab13970; 1:5000); tubulin (1:10,000) and ERK (1:10,000), diluted in 5% (w/v) skim milk (BD Difco) in TBS-T, followed by species-specific HRP-conjugated secondary antibodies (Jackson ImmunoResearch Laboratories; 1:10,000) and visualized using a myECL imager (Thermo Fisher Scientific), according to the manufacturer's instructions. Quantification was performed using ImageJ software.

##### Isolation and culture of human mesenchymal stem cells (hMSCs).

hMSCs from healthy donors and ALS patients used in this study were obtained from bone marrow samples and were isolated, and then phenotypically characterized and cultured as described previously (Nachmany et al., 2012). All volunteers in this work signed a consent form before sample donation, according to the guidelines of the Hospital's Ethics Committee supervised by the Israeli Health Ministry Ethics Committee conforming with the Code of Ethics of the World Medical Association (Declaration of Helsinki), printed in the *British Medical Journal* (Rickham, 1964).

##### MN cell culture.

Primary SC neurons were cultured using E12.5 mouse embryos of either sex as previously described (Zahavi et al., 2015). Briefly, SCs were excised, trypsinized, and triturated. Supernatant was collected and centrifuged through a 4% BSA cushion. The pellet was resuspended and centrifuged through an OptiPrep gradient (10.4% OptiPrep, Sigma-Aldrich; 10 mm Tricine, 4% glucose) for 20 min at 760 × *g* with the brake turned off. Cells were collected from the interface, washed once in complete medium, and then plated in coated growth chambers. Cells were maintained in Complete Neurobasal Medium (Invitrogen) containing B27 (Invitrogen), 10% (v/v) horse serum (Biological Industries), 25 nm β-mercaptoethanol, 1% penicillin-streptomycin (PS; Biological Industries), and 1% GlutaMAX (Invitrogen) supplemented with 1 ng/ml GDNF, 0.5 ng/ml CNTF, and 1 ng/ml BDNF (Alomone Labs). Before plating, the growth plates were coated with 1.5 g/ml poly-dl-ornithine (Sigma-Aldrich) overnight at 37°C and 3 g/ml laminin (Sigma-Aldrich) for 2 h at 37°C. For immunofluorescence staining, 30,000 cells were plated on cover slides in 24-well plates. Cells were grown at 37°C in 5% CO_2_.

##### SC explants.

SCs were dissected from E11.5 mouse embryos of both sexes, either using HB9::GFP or *SOD1^G93A^* stripped of meninges and DRGs. The ventral horn was separated from the dorsal horn by longitudinal cuts along the SC, and transverse sections up to 1 mm were placed in the explant well. Before plating, the growth chambers were coated with 1.5 g/ml poly-dl-ornithine overnight at 37°C and 3 g/ml laminin overnight at 37°C. Explants were maintained in Spinal Cord Explant Medium containing Neurobasal, 2% B27, 1% PS, and 1% GlutaMAX, supplemented with 25 ng/ml BDNF. Explants were grown at 37°C in 5% CO_2_.

##### Primary myocyte culture.

Skeletal muscle cultures were derived from the GC muscle of adult P60 female mice of either *SOD1^G93A^* background or their littermates (LMs) using techniques previously described (Ionescu et al., 2016). Briefly, GC muscles were excised and incubated in 2 mg/ml collagenase I (Sigma-Aldrich) in DMEM containing 2.5% PSN (Biological Industries) for 3 h. Muscles were then dissociated and incubated for 3 d in Matrigel-coated (BD Biosciences) 6-well plates with Bioamf-2 medium (Biological Industries) with 1% PSN at a density of ∼120 myofibers per well. For purification of the myoblasts, adherent cells were trypsinized and preplated in an uncoated dish for 1 h at 37°C. Nonadherent cells were then transferred into a Matrigel-coated dish with Bioamf-2 medium. Preplating was repeated for 2 d, keeping the culture at <50% confluence, before plating cells in MFC. Cultures were maintained at 37°C and in 5% CO_2_. After the final preplating, 100,000 myocytes were cultured in the precoated distal compartment of the MFC. Myocyte conditioned media (CM) were produced as follows: At the final preplating stage, myoblasts were cultured in a Matrigel-coated 100 mm dish at 80% confluence and were incubated for 2 d with Bioamf-2 medium, followed by 2 d with rich DMEM (Biological Industries) medium containing 10% FCS (Biological Industries), 10% horse serum (Biological Industries), 1% GlutaMAX, and 1% PSN. Then, once muscles reached a fully differentiated state, the culture dish was rinsed 3 times with preheated PBS and poor DMEM containing 1% GlutaMAX, and 1% PSN was applied on the cultures. CM was collected after 2 d, centrifuged for 5 min at 400 × *g* at 25°C, and streamed through a 0.22 μm PES filter.

##### CM preparation and application.

Muscle myocytes of WT or *SOD1^G93A^* mice were cultured as described previously (Ionescu et al., 2016). Seven days after myocytes were fully differentiated, the muscles kept growing for 3 d in complete Neurobasal containing BDNF and GDNF. The CM was refreshed with BDNF, GDNF, and glucose after its collection, as described previously (Nagai et al., 2007). CM under both conditions was applied on the axon compartment of the MFC for 48 h.

##### Lentiviral vectors.

Genes of interest were cloned into a third-generation lentiviral pLL3.7 backbone. HEK293T cells were transfected by using calcium phosphate method and a mixture consisting of the vector of interest, vesicular stomatitis virus glycoprotein, and group antigens-polymerase (reverse transcriptase) was used. The medium was replaced after 5–8 h, and the supernatant was collected 48 h later. Next, 50 mm HEPES was added before freezing to maintain a neutral pH for long-term storage. When necessary, lentiviruses were concentrated using a PEG Virus Precipitation Kit (Abcam).

##### NMJ staining.

GC was excised from P60 mice and cleared of connective tissue, washed in PBS, fixed in 4% PFA, washed once more, and then incubated with 1 g/ml Rhodamine Red-Conjugated Bungarotoxin (Sigma-Aldrich). Tissues were washed and then treated with methanol at −20°C for 5 min, washed, and then blocked in blocking solution for 1 h. Tissues were then rocked with appropriate primary antibodies diluted in blocking solution at room temperature overnight. Antibodies were used at the following concentrations: anti-Neurofilament Heavy Chain 1:500 (NFH, Abcam, ab72996; 1:1000), synaptophysin (Millipore, MAB5258; 1:300), synaptotagmin (Alomone Labs, ant-003; 1:300), anti-NRP1 (1:100), anti-Sema3A (1:100), anti-NRP2 (1:100), and anti-Sema3B (1:100). After having been washed, secondary antibodies (DyLight 405 anti-chicken 1:500; AlexaFluor-488 anti-chicken 1:500; AlexaFluor-647 anti-rabbit 1:500) were added for 4 h at room temperature. Muscle fibers were spread into monolayers under a stereomicroscope and affixed to slides using VectaShield (Vector Laboratories). Cover slides were sealed with clear nail polish.

##### Quantification of myocyte contraction.

The 1000-frame-long movies of myocytes in the distal compartment of the MFC were acquired 7 d after coculturing. Imaging was performed under bright-field conditions at a rate of ∼33 fps while using a 20× objective. A myocyte contraction plot was then profiled using an image-based method previously described (Zahavi et al., 2015; Ionescu et al., 2016). Briefly, only myocytes that came in contact with axons were plotted. Time-lapse images were taken for analysis using ImageJ. To create a time trace of contractions, high contrast (bright or dark) ROIs were selected on each myotube. Movement of the selected spot due to myotube contraction was assessed by the change in the ROI intensity over time. The number of strong contractions, as measured from the time trace, was manually validated by reexamining the time-lapse image series. The number of strong and weak contractions in innervated myotubes was compared before and after 1 μm TTX was added to the neuronal compartment. A myotube with a post- to pre-TTX difference of >50% was measured as an increase or decrease in contraction, and the fraction of increased, decreased, and unchanged myotubes was calculated.

##### Immunostaining of cell cultures.

Cultures were fixed in 4% PFA and permeabilized with 0.1% Triton X-100, 5% DS (Donkey Serum), 1 mg/ml BSA in PBS. Samples were blocked for 1 h with blocking medium containing 5% DS, 1 mg/ml BSA in PBS. Primary antibodies against NFH (1:500), NRP1 (1:100), Sema3A (1:100), NRP2 (1:100), Sema3B (1:100), and acetylated tubulin (1:1000), ryanodine receptor 1 (Millipore, AB9078; 1:500), α-actinin (Sigma-Aldrich, A5044; 1:400), Tau5 (Abcam, ab80579; 1:500) MAP2 (Millipore, ab5622; 1:500) were diluted in blocking solution and incubated overnight at 4°C. Samples were incubated with species-specific fluorescent secondary antibodies for 2 h at room temperature. DAPI was used for visualizing nuclei in myotubes. In MFC, after the staining protocol was completed, the MFC was peeled from the dish by gently pulling it from the proximal to the distal side. ProLong mounting medium was added, and samples were covered with a #1.5, 18 × 18 mm coverslide.

##### RNA extraction and cDNA synthesis.

Muscle tissues were immediately frozen with liquid nitrogen. Tissue was ground to powder using a pestle and mortar. Then 700 μl of TriReagent (Sigma-Aldrich) was added to the muscle powder, and the samples were further passed through a 21G needle 3 times for better homogenization. RNA from the TriReagent-rinsed samples was further isolated following the TriReagent protocol. RNA quality was measured using NanoDrop3000 and a bio-analyzer. RNA purification of MN mass culture, along with transfected HeLa cells, was performed using TriReagent protocol as well. mRNAs were pooled in equal amounts and reverse-transcribed into double-stranded cDNA by using the SuperScript2 kit (QIAGEN).

##### NanoString Chip.

A total of 100 ng RNA samples were outsourced to NanoString Technologies' facilities for an miR-Chip array assay of ∼800 known miRs (NanoString Technologies). miR was quantified automatically by NanoString Technologies' instrumentation for miRs, which was hybridized with the template. Output data were analyzed by the nCounter analysis system. All miRs were normalized to the 100 most abundant miRs in the samples.

##### Primer design.

Based on the consensus sequences of the desired transcripts, 2 sets of primers were designed for each gene (Table 1).

**Table 1.** Primer design

Gene	Forward primer	Reverse primer
*hHPRT*	GAACCAGGTTATGACCTTGATTTAT	GCAAGACGTTCAGTCCTGT
*hSema3A*	GCTCCAGTTATCATACCTTCCTTTTG	ACTGGCCACACAATCTTTTGAA
*hNRP1*	ACCTGTTCTCTTTCAGGGAA	CAAGTTGCAGGCTTGATTCG
*hB2M*	CCGTGTGAACCATGTGACTT	GGCATCTTCAAACCTCCATGA
*hNRP2*	GAGGCCAACCAGACCCA	CGTAAACAATCCACTCGCAGTT
*hSema3B*	TCTCCTTCCAAGTCCA	CTCGGCACCCACAAACA
*mSema3A*	CACTGGGATTGCCTGTCTT	GGCCAAGCCATTAAAAGTGA
*mGFP*	GCTACCCCGACCACATGAAGCA	GTCTTGTAGGTGCCGTCGTCCTTG
*m-miR126*	ID000451 (Thermo Fisher Scientific)	ID000451 (Thermo Fisher Scientific)

h, Human gene; m, murine gene.

##### qPCR for mRNA detection.

qPCR was performed on the StepOne system (Invitrogen) in a 10 μl reaction containing 4 μl of RNA (20 ng), 5 μl Syber green master mix (Thermo Fisher Scientific), and 1 μl of reverse and forward primers.

##### miR vectors and transfection.

Mammalian expression vector pMSCV-Blast-miR constructs were generously provided by Eran Hornstein (Weizmann Institute of Science). Mammalian expression vector of *C9orft72* Di-peptide PR_50_ and GR_50_ constructs was generously provided by David Trotti (Jefferson University) (Wen et al., 2014). Next, 50,000 HeLa/U87 human glioblastoma/muscles cells were plated in rich DMEM (1% PS, 1% GlutaMAX, 20% FBS). After 24 h, the culture medium was replaced with serum-free medium (Opti-MEM), and cells were transfected using FuGene NE 6 (Promega) protocol. Cells were collected after 48 h and used either for a functional assay or for RNA/protein extracts. Myocyte cultures were transfected using the same approach.

##### Semaphorin preparation.

HEK293T cells were stably transfected to overexpress either Sema3A or an empty control. CM from 80% confluent cultures were collected after 3 d. We validated the purity level of the collected media using Coomassie staining and identified the stained band with a specific antibody against the desired protein using Western blot analysis.

##### NRP1 antibody application.

A total of 5 μg/ml NRP1 antibody (R&D System, AF566 dot ETH0915031) for the extracellular domain was added to the distal compartment of the MFC while maintaining a proximal-to-distal volume gradient.

##### Histology tissue collection and fixation.

GC muscles of 20 samples were harvested and fixed in 4% PFA. The samples were then outsourced for a histological assessment at Patho-Logica. All tissues were trimmed into block cassettes and sent to CDX Diagnostics for slide preparation.

##### Slide preparation and histological evaluation.

Tissues were trimmed, embedded in paraffin sections at no more than 5 μm thickness, and stained with H&E. The mean minimal muscle fiber diameter thickness was measured in microns by performing a manual count using a 10× lens and analyzed by expert pathologist.

##### xCELLigence impedance measurement.

For each experiment, 30,000 U87 cells were plated with rich DMEM in E-Plate L8 wells and incubated together with the xCELLigence system (ACEA Biosciences) at 37°C, 5% CO_2_ overnight. Impedance data were collected at 5 min intervals. After 24 h, poor DMEM (1% PS, 1% GlutaMAX) with Sema3A or its control medium was replaced and recording proceeded. The data were analyzed using RTCA data analysis software 1.0 and normalized to the control sample.

##### CatWalk XT gait analysis.

The CatWalk is a video-based analysis system used to assess gait in voluntarily walking mice (Noldus Information Technology). The principle of this method is based on an optical technique. The light of a fluorescence tube is completely internally reflected on a glass walkway floor. When the animal crosses the walkway, the light leaves the glass and illuminates only the area of contact. In this way, the different paw contacts are visualized. Based on position, pressure, and the surface area of each foot paw, multiple parameters are calculated. Only compliant and continuing trials for each animal were analyzed, averaged, and the mean was calculated.

##### Experimental design and statistical analysis.

Data are mean ± SEM. The statistical analysis was assessed by Student's *t* test. In all cases, differences were considered to be statistically significant if *p* < 0.05.

## Results

### Sema3A and NRP1 levels are elevated in muscles and the MNs of ALS models

**Figure 1.**
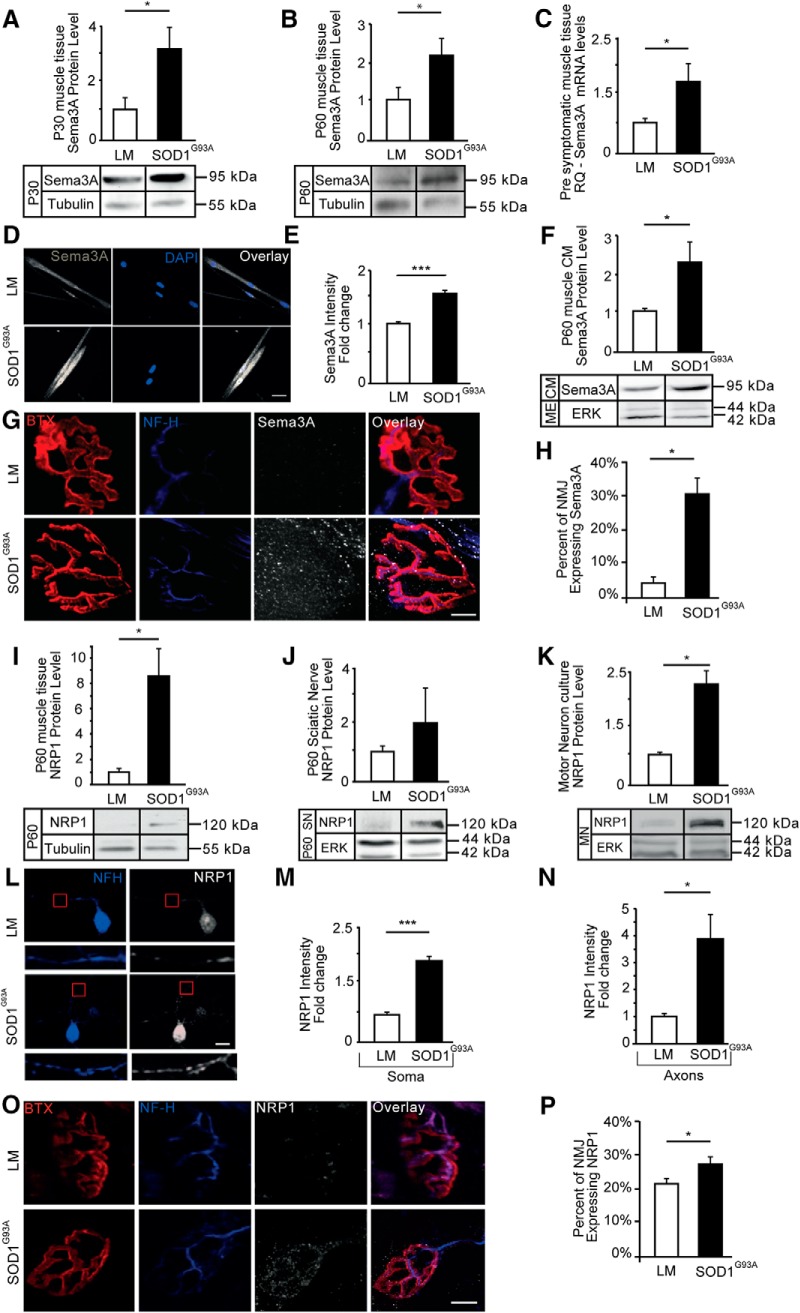
Presymptomatic elevation in the levels of Sema3A and NRP1 in ALS models. ***A***, ***B***, Western blot analysis of P30 and P60 GC muscle extracts revealed that the levels of Sema3A are elevated in presymptomatic *SOD1^G93A^* muscles compared with their corresponding LM control wherein at earlier stages we found no significant difference (Figure 1-1). Tubulin was used as a loading control. P30 (Student's *t* test, *n* = 3, *p = 0.042). P60 (Student's *t* test, *n* = 4, *p = 0.038). ***C***, qPCR analysis of presymptomatic P60 and P30 GC muscle extracts also shows an elevation in the mRNA levels of Sema3A in *SOD1^G93A^* (Student's *t* test, *SOD1^G93A^*, *n* = 5, LM, *n* = 4, *p = 0.049). ***D***, Immunostaining of primary myocytes after 7 d in culture shows elevated levels of Sema3A in primary myocytes of *SOD1^G93A^*. White represents Sema3A. Blue represents nuclear DAPI staining. Scale bars, 5 μm. ***E***, Image analysis reveals an increase in Sema3A intensity in *SOD1^G93A^* primary myocytes (Student's *t* test, *n* = 3, ∼80 cells, ***p = 0.00001). ***F***, Western blot analysis of primary myocyte-CM revealed a higher level of Sema3A in the CM of *SOD1^G93A^*. Cultures were lysed after CM collection, and equal loading volumes of lysates were immunoblotted for ERK to validate CM, which was produced from a similar mass of myocytes (Student's *t* test, *n* = 3, *p = 0.018). ***G***, ***H***, Immunostaining of fixed whole P60 GC muscles shows distinct Sema3A expression in the NMJs of *SOD1^G93A^* mice. White represents Sema3A. Red represents TMR-BTX labeling of acetylcholine receptors on postsynapse. Blue represents presynaptic NFH in neurons. The percentage of muscle fibers expressing Sema3A in their NMJs in P60 *SOD1^G93A^* mice is higher (∼100 NMJ per 1 biological repeat; Student's *t* test, *SOD1^G93A^*, *n* = 4; WT, *n* = 3; *p = 0.011). Scale bars, 10 μm. We also examined Sema3A expression in later stages of the disease (Figure 1-2). ***I***, Western blot analysis of GC muscle extracts from P60 mice revealed elevated NRP1 levels in the muscles of *SOD1^G93A^*. Tubulin was used as a loading control (Student's *t* test, *n* = 3, *p = 0.048). ***J***, Western blot analysis of SN extract from P60 mice shows an elevation in the levels of NRP1 in the SNs of *SOD1^G93A^* mice (*n* = 3). ***K***, Western blot analysis of primary MN lysates after 3 d in culture reveals an elevation in the NRP1 levels in *SOD1^G93A^* MNs, which are not regulated by Sema3A binding (Figure 1-3). ERK was used as a loading control (Student's *t* test, *n* = 3, *p = 0.031). ***L–N***, Immunostaining of primary MNs after 3 d in culture shows an elevation in the levels of NRP1 in both axons (inset, ∼4.1-fold) and somata (∼1.9-fold) of *SOD1^G93A^* MNs. White represents NRP1. Blue represents NFH. Somata (Student's *t* test, *n* = 3, ∼40 cells, ***p = 0.00021); axons (Student's *t* test, *n* = 3, ∼40 cells, *p = 0.012). Scale bars, 10 μm. ***O***, ***P***, Immunostaining of fixed whole P60 GC muscles shows distinct NRP1 expression in the NMJs of *SOD1^G93A^* mice. White represents NRP1. Red represents BTX. Blue represents NFH. The percentage of muscle fibers expressing NRP1 in their NMJs in P60 *SOD1^G93A^* mice is higher (Student's *t* test, *SOD1^G93A^*, *n* = 4; WT, *n* = 3; *p = 0.042). Scale bars, 5 μm. We further examined NRP1 expression in later stages of the disease (Figure 1-2). Elevations in Sema3A and its coreceptor were found also in human sALS samples (Figure 1-4). ***A–C***, ***E***, ***F***, ***I–K***, ***M***, ***N***, Data represent the mean fold difference over the LM control ± SEM.

**Figure 2.**
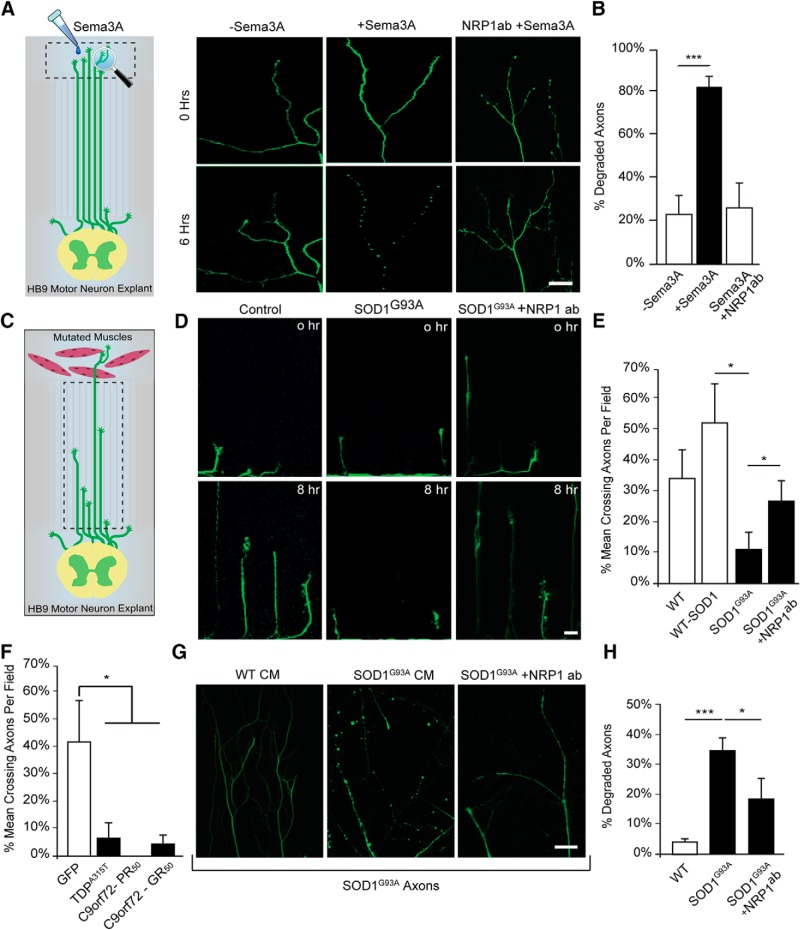
Sema3A as well as primary myocytes expressing diverse ALS-causing mutations impair the growth of wild-type HB9::GFP motor axons and enhance their retraction and degeneration. ***A***, Experimental procedure illustration and representative time-lapse images of HB9::GFP motor axons (Figure 2-1) in the distal compartment of an MFC with no muscles after applying Sema3A to the distal compartment. After 6 h, axons in the distal compartment of chambers that were treated with Sema3A undergo degeneration, whereas axons in the control chamber or axons cotreated with NRP1 antibody and Sema3A continue growing. Scale bar, 20 μm. ***B***, Quantification of the rate of degraded axons in the distal compartment revealed a higher percentage of degradation in chambers that were exposed to Sema3A compared with either control or coapplication of Sema3A and NRP1 antibody (∼60 axons for Sema3A treatment, ∼70 axons for Control; Student's *t* test; *n* = 4; mean ± SEM, ***p = 0.00022). ***C***, Schematic view of the experimental procedure in ***D–F***. HB9::GFP SC explants and primary myocytes of *SOD1^G93A^*, *TDP43^A315T^*, *C9orf72-PR_50_*, *C9orf72-GR_50_*, or LM, GFP, and SOD1^wt^ as controls were cocultured in an MFC (Figure 2-2), and the growth of HB9::GFP axons was assessed by time-lapse imaging of the microgroove compartment. ***D***, Representative time-lapse images of the HB9::GFP axon growth when cocultured with (left to right) LM, *SOD1^G93A^*, and *SOD1^G93A^* + NRP1 antibody. The presence of *SOD1^G93A^* myocytes in the distal compartment triggers the retraction and degeneration of HB9::GFP motor axons growing in the groove compartment and prevents their traversing. When NRP1 antibody is applied to the distal compartment, together with *SOD1^G93A^*-expressing myocytes, axons are less prone to degenerate. Scale bar, 5 μm. ***E***, Quantification of the rate of axons traversing the distal compartment in ***B*** shows the mean percentage of axons that traversed the distal compartment out of the total axons in each field (*n* = 3; NRP1 antibody experiment, *n* = 4; Student's *t* test, *p = 0.025, *p = 0.0433). ***F***, Quantification of the rate of axons traversing the distal compartment shows the mean percentage of axons that traversed the distal compartment out of the total number of axons in each field in coculture with TDP43^A315T^, C9orf72-PR_50_, C9orf72-GR_50_ myocytes, or GFP as a control. The traversing rate of HB9::GFP motor axons into the distal compartment in each of the cocultures with muscle-expressing ALS mutations is significantly reduced (*n* = 3; Student's *t* test, GR_50_, *p = 0.0137; PR_50_, *p = 0.0374; TDP, *p = 0.0304). ***G***, Representative images of fixed and immunostained *SOD1^G93A^* motor axons in the distal compartment of an MFC after applying LM or *SOD1^G93A^* myocyte CM to the distal compartment. After 48 h, axons in the distal compartment of chambers that were treated with *SOD1^G93A^* CM underwent degeneration, whereas axons that were treated with LM CM remained intact. WT MN axons remained intact after application of either CM (Figure 2-3). When NRP1 antibody is applied to the distal compartment, together with *SOD1^G93A^* CM, axons are less prone to degenerate, suggesting the involvement of multiple factors (Figure 2-4). Green represents acetylated tubulin. Scale bar, 20 μm. ***H***, Quantification of the rate of degenerated *SOD1^G93A^* axons in the distal compartment treated with control CM, *SOD1^G93A^* CM, or *SOD1^G93A^* CM that was coincubated with anti-NRP1 antibody (Student's *t* test; *n* = 3; ***p = 5 × 10^−7^, *p = 0.018). **p < 0.01.

**Figure 3.**
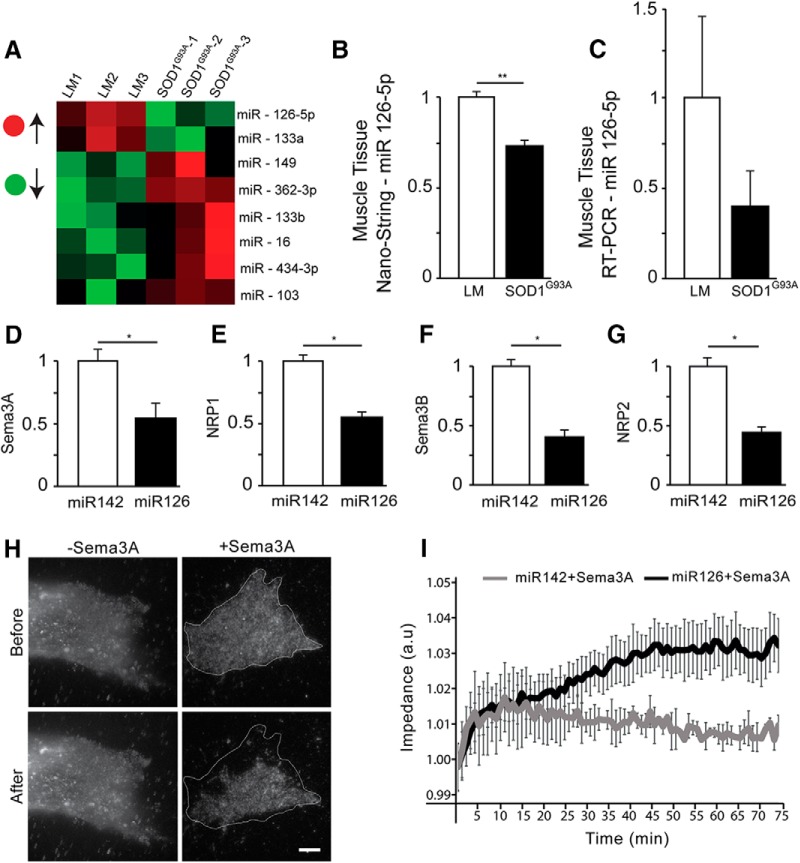
miR126-5p is depleted in *SOD1^G93A^* muscles and regulates Sema3 and NRP expression. ***A***, NanoString chip screen heat map of significantly altered miRs in P60 muscles of *SOD1^G93A^* compared with LM mice (extended table under Figure 3-1). Red and green represent a high or low abundance of miRs, respectively. *p < 0.05 (Student's *t* test; *n* = 3). ***B***, miR126-5p was the most significantly downregulated miRNA in *SOD1^G93A^* muscles (Student's *t* test; *n* = 3, **p = 0.003). ***C***, qPCR analysis of P60 GC muscle extracts further validates the decrease in miR 126-5p in *SOD1^G93A^* (*n* = 3). ***D–G***, qPCR analysis of Sema3A, NRP1, Sema3B, and NRP2 transcript levels in HeLa cells overexpressing either miR126-5p or miR142 demonstrates a reduction in their expression levels specifically under miR126-5p overexpression (Student's *t* test; *n* = 3, *p = 0.0438, *p = 0.034, *p = 0.031, and *p = 0.0434, respectively). ***H***, Representative TIRF images of U87MG cells reveal a detachment of the cell membrane from the culture dish surface after Sema3A is added to the culture medium. Scale bar, 10 μm. ***I***, Impedance recording of live cells over time shows that U87MG cells overexpressing miR126-5p are unresponsive to Sema3A added to the culture medium because their impedance continuously increases, whereas the impedance of U87MG cells overexpressing miR142 decreases after treatment (Figure 3-2).

**Figure 4.**
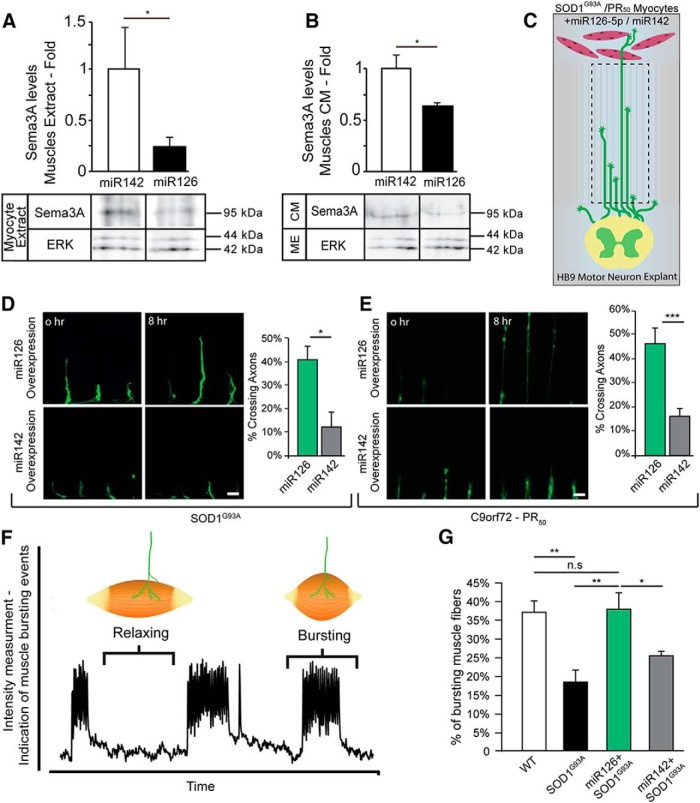
Overexpression of miR126-5p in primary *SOD1^G93A^* myocytes blocks motor axon degeneration and preserves NMJ activity in a compartmental coculture. ***A***, ***B***, Western blot analysis of transfected myocyte extract overexpressing miR126-5p or miR142 and their CM validates miR126-5p as a regulator of Sema3A specifically in muscles. ERK was used as a loading control (Student's *t* test, *n* = 3, *p = 0.0499 and *p = 0.05, respectively). ***C***, Schematic view of the experimental procedure in ***D***, ***E***. HB9::GFP SC explants and primary myocytes of *SOD1^G93A^* mice were cocultured in a MFC. The growth of the HB9::GFP axons was assessed both by time-lapse imaging of the microgroove compartment and by imaging axons that traversed the distal compartment. ***D***, Representative time-lapse images and quantification of HB9::GFP axon growth when cocultured with SOD1^miR126^ myocytes (top) or SOD1^miR142^ myocytes (bottom). SOD1^miR126^ myocytes in the distal compartment enhanced the axonal traversal of the distal compartment compared with the SOD1^miR142^ myocytes. The data are shown as the mean rate of axons that traversed the distal compartment out of the total number of axons in each field ± SEM (Student's *t* test, *n* = 3, *p = 0.04216). ***E***, Representative time-lapse images and quantification of HB9::GFP axon growth when cocultured with PR_50_^miR126^ myocytes (top) or PR_50_^miR142^ myocytes (bottom). PR_50_^miR126^ myocytes in the distal compartment enhanced the axonal traversal of the distal compartment compared with PR_50_^miR142^ myocytes. The data are shown as the mean rate of axons that traversed the distal compartment out of the total number of axons in each field ± SEM (Student's *t* test, *n* = 3, ***p = 0.0039). ***F***, Representative myocyte contraction plot showing the bursting contractile behavior of innervated myocytes *in vitro*. ***G***, Quantification of the percentage of innervated myocytes that contract in a bursting pattern shows a diminished rate of bursting behavior in *SOD1^G93A^* myocytes compared with LM controls. SOD1^miR126^ myocytes show an increase in the rate of bursting myocytes back to the LM levels. The data are shown as the mean percentage of bursting myocytes ± SEM (Student's *t* test, *n* = 3, *p = 0.0291, *p = 0.0156, **p = 0.005656). ***p < 0.001.

ALS disease is considered to be a distal axonopathy involving axon degeneration and NMJ disruption as key processes in its pathology (Fischer et al., 2004). We therefore hypothesized that destabilizing factors secreted from adult presymptomatic ALS mutant muscles might be involved in triggering axon degeneration of MNs. Because Sema3A is known to act in such a manner, at least in development, and it was already reported to be elevated in ALS, we decided to focus on this factor (De Winter et al., 2006; Körner et al., 2016). Following this hypothesis, we first examined the expression of Sema3A in *SOD1^G93A^* GC muscles in comparison with that of their LMs; (Fig. 1*A*,*B*; Fig. 1-1). Western blot analysis of muscle protein extracts revealed significant elevations in Sema3A protein levels in muscles of presymptomatic *SOD1^G93A^* mice as early as P30 and P60, whereas testing Sema3A levels in younger animals (P7) showed no apparent differences compared with their LM controls (mean fold change over LM: P30 *SOD1^G93A^*, 3.08 ± 0.86; P30 LM, 1 ± 0.36; P60 *SOD1^G93A^*, 2.2 ± 0.45; P60 LM, 1 ± 0.32; P7 *SOD1^G93A^*, 1.2 ± 0.7; P7 LM, 1 ± 0.42). To validate this difference, we also tested the transcript levels of Sema3A (Fig. 1*C*). qPCR analysis of total RNA extracts from muscles at presymptomatic *SOD1^G93A^* stage and LM mice identified an ∼1.7-fold increase in Sema3A mRNA of *SOD1^G93A^* muscles (mean fold change over LM: *SOD1^G93A^*, 1.72 ± 0.32; LM, 1 ± 0.06). Because GC muscle tissues contain heterogeneous cell types and to verify that the levels of Sema3A are indeed higher specifically in *SOD1^G93A^* muscles fibers, we immunostained primary myocyte cultures from P60 *SOD1^G93A^* and LM mice for Sema3A (Fig. 1*D*,*E*). Quantifying the mean intensity values showed a significant 50% increase in the *SOD1^G93A^* myocytes (a mean fold change in intensity over LM: *SOD1^G93A^*, 1.5 ± 0.06; LM, 1 ± 0.04). We also collected CM from myocyte cultures to determine whether the increase in Sema3A protein also results in an increase in its secretion (Fig. 1*F*). Western blot analysis indicated that Sema3A levels were also elevated in P60 *SOD1^G93A^* myocyte-CM (a mean fold change over LM: *SOD1^G93A^*, 2.3 ± 0.55; LM, 1 ± 0.08). Because NMJ disruption is a primary event in ALS, we sought to examine the levels of Sema3A in NMJ *in vivo* (Fig. 1*G*,*H*). Immunostaining for Sema3A in GC muscles showed a sixfold increase in the number of muscle fibers expressing Sema3A in their NMJs. Whereas only ∼5% of NMJs stained positively for Sema3A in LM muscles, we identified its expression in ∼30% of NMJs in P60 *SOD1^G93A^* mice (the mean percentage of NMJs expressing Sema3A: *SOD1^G93A^*, 30.83 ± 4.73%; LM, 4.56 ± 2.4%). Interestingly, a previous study described Sema3A elevation in SOD^G93A^ mice specifically in fast fatigue NMJs expressing myosin-IIb marker (De Winter et al., 2006). Because fast fatigue NMJs are the first to become disrupted and be eliminated in ALS pathology, we examined Sema3A levels both at P90 and P120 and hypothesized that Sema3A levels will eventually drop in later stages of the disease. We found that whereas the percentage of NMJs expressing Sema3A in SOD^G93A^ in P90 animals is similar to P60, the end-stage animals (P120) were shown to display a reduction in Sema3A-positive NMJs, and no apparent difference existed between WT and *SOD1^G93A^* mice (Fig. 1-2*A*,*B*). Together, these results indicate that a significant part of the MN population is exposed to high levels of Sema3A in presymptomatic stages and that this specific population is disrupted and eliminated during disease progression. We then proceeded to investigate the expression of the Sema3A-receptor binding unit, NRP1, in ALS (Fig. 1*I*). Western blot analysis of GC muscle extracts revealed a significant ∼8-fold increase in NRP1 (the mean fold change over LM: *SOD1^G93A^*, 8.6 ± 2.2; LM, 1 ± 0.3). Because MNs are a primary target in ALS, we wanted to determine whether NRP1 is also overexpressed in the MNs of *SOD1^G93A^* mice. First, we performed Western blot analysis of SNs and observed an ∼2-fold elevation in NRP1 levels of P60 *SOD1^G93A^* mice (Fig. 1*J*; the mean fold change over LM: *SOD1^G93A^*, 1.96 ± 1.22; LM, 1 ± 0.21). Next, we obtained protein extracts of primary MN cultures for Western blot analysis and confirmed an ∼2.5-fold elevation in NRP1 levels in the MNs of *SOD1^G93A^* culture (Fig. 1*K*; the mean fold change over LM: *SOD1^G93A^*, 2.3 ± 0.16; LM, 1 ± 0.06). Immunostaining of primary MN cultures for NRP1 resulted in analogous findings (Fig. 1*L–N*). Intriguingly, the NRP1 signal in *SOD1^G93A^* is generally higher than in LM and is increased even more in axons compared with cell bodies (the mean fold change over LM: *SOD1^G93A^* soma, 1.86 ± 0.13; LM soma, 1 ± 0.05; *SOD1^G93A^* axon, 3.83 ± 0.95; LM axon, 1 ± 0.11). Finally, immunostaining for NRP1 in GC muscles confirmed a similar shift of ∼30% in the number of NMJs expressing NRP1, as we had observed for Sema3A in *SOD1^G93A^* mice, both at P60 and P90. However, also this time, the differences were abolished in the end stages of the disease (P120) (Fig. 1*O*,*P*; Fig. 1-2*C*,*D*; the mean percentage of NMJs expressing NRP1: P60: *SOD1^G93A^*, 27.5 ± 2.04%; LM, 21.27 ± 1.22%). To determine whether the elevated NRP1 levels result from feedback due to an increase in its ligand, we treated primary MN cultures from LM embryos with soluble Sema3A for 3 d and performed Western blot analysis on cell culture lysates. Importantly, we did not observe any difference in NRP1 expression after applying Sema3A, suggesting that NRP1 levels are regulated by an intrinsic mechanism in MNs (Fig. 1-3). Finally, to validate our finding with other ALS models and to emphasize the impact of Sema3A in ALS, we performed Western blot analysis for Sema3A and NRP1 expression in human mesenchymal stem cells from sporadic ALS patients and healthy controls, as well as in myocyte-expressing C9orf72-PR_50_ and their CM for Sema3A. In addition, we compared the results with those of a mock control. Remarkably, in all of these ALS models, we identified high expression of Sema3A and NRP1 (Fig. 1-4).

10.1523/JNEUROSCI.3037-17.2018.f1-1 Figure 1-1 **Sema3A protein levels in P7 SOD1^G93A^ GC muscle.** (A) Western blot analysis of P7 GC muscle extracts reveals that the levels of Sema3A (∼95 kDa) are similar in pre-mature SOD1^G93A^ muscles compared with their corresponding LM control. ERK (-42-44 kDa) was used as a loading control. (The mean fold change over control: SOD^G93A^ 1.2 ± 0.74; LM 1 ± 0.42; Student’s t-test, n=3, n=2, respectively; n.s). Download Figure 1-1, TIF file

10.1523/JNEUROSCI.3037-17.2018.f1-2 Figure 1-2 **Sema3A and NRP1 expression in the NMJs of SOD^G93A^ mice at the age of P90 and P120.** (A-B) The percentage of muscle fibers expressing Sema3A in their NMJs in P90 SOD1^G93A^ mice is higher than in LM (SOD^G93A^ 26.92% ± 5.07%; LM 8% ± 1.36% (∼100 NMJ per 1 biological repeat; Student’s t-test, SOD1^G93A^ n=3, WT n=3, *p=0.023982), whereas in P120 mice this difference was abolished (SOD^G93A^ 10.22% ± 2.61%; LM 9.89% ± 2.21% (∼100 NMJ per 1 biological repeat; Student’s t-test, SOD1^G93A^ n=3, WT n=3, n.s). (C-D) The percentage of muscle fibers expressing NRP1 in their NMJs in P90 SOD1^G93A^ mice is higher than in LM (SOD^G93A^ 29.17% ± 1.86%; LM 21.66% ± 2.09% (∼100 NMJ per 1 biological repeat; Student’s t-test, SOD1^G93A^ n=3, WT n=3, *p=0.02767), whereas in P120 mice this difference was abolished (SOD^G93A^ 14.26% ± 2.43%; LM 18.14% ± 1.67% (∼100 NMJ per 1 biological repeat; Student’s t-test, SOD1^G93A^ n=3, WT n=3, n.s). Download Figure 1-2, TIF file

10.1523/JNEUROSCI.3037-17.2018.f1-3 Figure 1-3 **NRP1 levels in MN are not regulated by Sema3A binding.** (A) Western blot analysis of wild-type MN that were cultured in the presence or absence of Sema3A for 3 days indicate no alterations in the levels of NRP1 (120kDa) in the Sema3A-treated group. ERK (42-44 kDa) was used as a loading control (the mean fold difference in NRP1 levels over control treatment +Sema3A 0.78 ± 0.1; -Sema3A 1 ± 0.16; n=4, n.s). Download Figure 1-3, TIF file

10.1523/JNEUROSCI.3037-17.2018.f1-4 Figure 1-4 **Sema3A and NRP1 elevations in human sALS patients and C9orf72-PR_50_ mutant myocytes.** (A) Western blot analysis of hMSC lysate from sALS patients and healthy controls indicates Sema3A (∼95 kDa) elevation in human patients. ERK (42-44kDa) was used as a loading control (the mean fold change over controls: sALS 1.3 ± 0.22; healthy controls 1 ± 0.2, n=4). (B) Western blot analysis of hMSC lysates from sALS patients and healthy controls indicates similar elevations of NRP1 (120kDa) in human patients. ERK (42-44 kDa) was used as a loading control (the mean fold change over control: sALS 5.7 ± 1.5; healthy controls 1 ± 0.4; Student’s t-test, n=4; *p=0.01). (C) Western blot analysis of primary myocyte culture extract reveals a higher level of Sema3A in C9orf72-PR_50_ mutant muscles compared with the m.Cherry control. (The mean fold change over controls: PR_50_ 2.89 ± 1; m.Cherry 1 ± 0.357, Student’s t-test, n=3; *p=0.049.). (D) Western blot analysis of primary myocyte culture-conditioned media of the muscle used in C reveals a higher level of Sema3A in conditioned media of C9orf72-PR_50_ mutant muscles over control. (The mean fold change over controls: PR50 4.45 ± 1.37; m.Cherry 1 ± 0.3, Student’s t-test, n=3; *p=0.029.). Download Figure 1-4, TIF file

Together, our combined *in vivo* and *in vitro* data suggest that the levels of both Sema3A and its cobinding receptor, NRP1, are presymptomatically increased in several ALS models as well as in sALS patients. These findings suggest that the Sema3A pathway is a common denominator in various ALS mutations; thus, it may contribute to MN degeneration in ALS.

### Application of Sema3A on wild-type MN axons results in axon degeneration

Because our findings suggest that Sema3A is produced and secreted in excess from muscles of ALS models, and because muscles interact specifically with MN axons, we sought to test the activity of Sema3A exclusively in this distal subcellular compartment. To this end, we used an MFC that allows precise control, monitoring, and manipulation of subcellular microenvironments (Fig. 2-1) (Zahavi et al., 2015). We cultured healthy ventral SC explants from transgenic mouse embryos expressing GFP under the MN-specific promoter HB9 (HB9::GFP) in one compartment of the MFC and enabled axons to extend into the opposing compartment, thus segregating axons and cell bodies into two isolated compartments. To verify that our MFCs can efficiently segregate MN axons from their somata, we stained the neuronal culture in the MFC system for the dendritic and axonal markers MAP2 and Tau, respectively (Fig. 2-1). We confirmed that all neurites that traversed the distal compartment are positive for Tau staining and negative for MAP2. Next, we purified Sema3A or control media as described previously (Ben-Zvi et al., 2006), and applied them to the distal compartment, while imaging the axons for 16 h (Fig. 2*A*). Our recordings reveal extensive axon degeneration in the Sema3A-treated MFCs 6–8 h after its application (Fig. 2*B*; Movies 1, 2; the mean percentage of degenerated axons: Sema3A, 83.01 ± 3.54%; control, 23.94 ± 7.6%). Coapplication of NRP1-blocking antibody and Sema3A on MN axons inhibits the Sema3A-dependent axon degeneration (Fig. 2*B*; the mean percentage of degenerated axons: Sema3A and NRP1 antibody, 25.00 ± 12%). These data indicate that Sema3A can trigger axon degeneration in MNs when applied exclusively on distal axons, and further support our hypothesis that an increase in muscle-derived Sema3A might contribute to axon degeneration in ALS.

10.1523/JNEUROSCI.3037-17.2018.f2-1 Figure 2-1 **Micro-Fluidic-Chamber efficiently separates the distal axons from the proximal cell bodies and dendrites.** (A) Simplified illustration of the compartmentalized microfluidic chamber used to culture spinal cord explants and primary myocytes in two different compartments connected via parallel microgrooves. (B) Representative images of HB9::GFP motor axons co-cultured in the presence (left panel) or absence (right panel) of wild-type primary myocytes in a microfluidic chamber, showing that myocytes facilitate the directed traversal of HB9::GFP motor axons into the distal compartment. Scale bar, 100µm. (C) Quantitative analysis of the axonal traversal rate per chamber of HB9::GFP explant cultured in the presence or absence of myocytes, which shows a significant increase in the traversal of axons when myocytes are present in the distal compartment (the mean number of traversing axons per chamber after 3 days in culture: with myocytes 8.5±1.5; no myocytes 2.3±0.42 **P=0.0026; Student's t-test; n=6). (D) Immunostaining of a motor neuron culture in a MFC system for MAP2, TAU, and DAPI markers revealed that all neurites that traverse the distal side of the chambers are positive for TAU (axonal marker) and negative for MAP2 (dendritic marker). Red indicates MAP2, green indicates TAU, and blue indicates DAPI. Scale bar: 200 µm. Download Figure 2-1, TIF file

10.1523/JNEUROSCI.3037-17.2018.f2-2 Figure 2-2 **Manipulated myocyte cultures showing no morphological differences compared to healthy ones.** (A) Representative images of all of the following *in vitro* primary cultured myocytes (top to bottom) WT, SOD1^G93A^, TDP43^A315T^, C9orf72-PR_50_, C9orf72-GR_50_, miR126-5p, and miR142 at 12 DIV. Under all muscle culture conditions, similarity in morphology, fusion, and health was observed. Blue indicates DAPI. Scale bar: 50µm. (B) Quantitative analysis of the mean nuclei per myocyte for each of the described conditions (the mean fold difference over the WT control ± sem: WT 1±0.09, SOD1^G93A^ 1.1±0.08, TDP43^A315T^ 0.94±0.07, C9orf72-GR_50_ 0.97±0.05, C9orf72-PR_50_ 1.16±0.06, miR126-5p 1.16±0.06, m5R142 1.5±0.07. (C) Immunostaining of LM and SOD1^G93A^ primary myocytes revealed a similarity in differentiation under both conditions at 19 DIV. Nuclear DAPI staining shows peripherally distributed nuclei. Intensity measurements of both alpha-actinin and Ryanodine Receptor (RyR) revealed an opposing stripe-like structure indicating their maturation. Red indicates alpha-actinin, green indicates RyR , blue indicates DAPI. Scale bar: 200 µm. Download Figure 2-2, TIF file

10.1523/JNEUROSCI.3037-17.2018.f2-3 Figure 2-3 **WT and SOD^G93A^ conditioned media applied on WT axons.** (A) Representative images of fixed and immunostained WT motor axons in the distal compartment of a microfluidic chamber after applying LM or SOD1^G93A^ myocytes (CM) to the distal compartment. After 48 hours, axons in the distal compartment of chambers that were treated with either SOD1^G93A^ or WT CM remain intact. Green indicates Acetylated Tubulin. Scale bar, 20µm. (B) Quantification of the rate of degenerated WT axons in the distal compartment treated with WT CM or SOD1^G93A^ CM (the mean percentage of degenerated axons per field: SOD1^G93A^ 6.88% ± 1.7%; WT 7.47% ± 1.22%). Download Figure 2-3, TIF file

10.1523/JNEUROSCI.3037-17.2018.f2-4 Figure 2-4 **Sema3B and NRP2 levels are elevated in the SOD1^G93A^ ALS mouse model as well as in human sALS patients.** (A-B) Immunostaining of fixed whole P60 GC muscles shows distinct Sema3B expression in the NMJs of SOD1^G93A^ mice. Green indicates Sema3B, red indicates postsynaptic Acetylcholine receptors labeled with BTX and blue indicates NFH in presynaptic neurons. The percentage of NMJs expressing Sema3B in P60 muscles is higher in SOD1^G93A^ mice. **p<0.01 (Student’s t-test, n=3) (the mean percentage of NMJs expressing Sema3B: SOD1^G93A^ 49.3% ± 1%; LM 25% ± 2.5%). Scale bars: 5µm. (C-D) Immunostaining of fixed whole P60 GC muscles shows distinct NRP2 expression in the NMJs of SOD1^G93A^ mice. Green indicates NRP2, red indicates postsynaptic Acetylcholine receptors labeled with BTX, and blue indicates NFH in presynaptic neurons. The percentage of NMJs expressing NRP2 in P60 muscles is higher in SOD1^G93A^ mice. **p<0.01 (Student’s t-test, n=3) (the mean percentage of NMJs expressing NRP2: SOD1^G93A^ 50% ± 2%; LM 33.5% ± 4.5%). Scale bars: 5µm. Data show the mean fold difference over LM control ±SEM. Download Figure 2-4, TIF file

Movie 1. Sema3A control medium on HB9::GFP distal axons. Time-lapse image series of HB9::GFP axons in the distal compartment of an MFC with control medium added to the distal compartment. Scale bar, 50 μm. 10.1523/JNEUROSCI.3037-17.2018.video.1

Movie 2. Sema3A in the distal compartment drives the degeneration of HB9::GFP distal axons. Time-lapse image series of HB9::GFP axons in the distal compartment of an MFC with Sema3A added to the distal compartment. Scale bar, 50 μm. 10.1523/JNEUROSCI.3037-17.2018.video.2

### Muscles expressing diverse ALS mutations initiate axon degeneration

To study the molecular mechanisms enabling the communication between MNs and their environment, which are essential for cell survival and synapse maintenance, we extended the use of the MFC system to coculture primary MNs and primary myocytes (Ionescu et al., 2016). Briefly, ventral SC explants from healthy 12-day-old (E12) HB9::GFP embryos were cultured in the proximal compartment, in the presence or absence of primary myocytes extracted from adult mice in the distal compartment (Fig. 2-1). As we showed previously (Zahavi et al., 2015), culturing HB9::GFP explants in the presence of wild-type muscles facilitates the rapid and directed growth of axons into the distal compartment (Fig. 2-1), suggesting that muscles secrete factors that support and promote the growth of motor axons. However, because ALS-mutated muscles were found to have intrinsic abnormalities throughout disease progression (Loeffler et al., 2016), we hypothesized that the nature of these factors will be altered. To study the effect of ALS muscles on MN axons in a simplified system, we plated primary myocytes from presymptomatic P60 *SOD1^G93A^* and LM mice as well as WT myocytes transfected to express SOD1^wt^ in the distal compartment. Myocyte cultures were allowed to fuse and differentiate. Importantly, in all the described cases, myocyte morphology, fusion, and differentiation parameters were similar, and the culture showed no apparent difference (Fig. 2-2). After 7 d, HB9::GFP SC explants were cultured in the proximal compartment. Cocultures were incubated until the HB9::GFP axons began extending toward the microgroove compartment. Once the axons reached the microgroove compartment, the extension of HB9::GFP axons along the microgrooves was recorded for 16 h (Fig. 2*C*). Surprisingly, HB9::GFP axons that were cocultured with the *SOD1^G93A^* myocytes were less likely to traverse the distal side (Fig. 2*D*,*E*). During this period, axons extending toward the *SOD1^G93A^* myocytes were markedly incapable of traversing the distal compartment and underwent retraction, degeneration, or remained static in place, compared with the LM and SOD1^wt^ controls (Fig. 2*D*; Movies 3, 4). Noteworthy, the addition of NRP1-blocking antibodies to the distal compartment, targeting Sema3A binding to the extracellular site of NRP1, improved the traversing rate of axons (Fig. 2*E*; the mean axon traversal rate per field: LM, 33.88 ± 10.40%; SOD1^wt^, 52.66 ± 12.7% *SOD1^G93A^*, 11.1 ± 5.5%; SOD1^G93A+NRP1-ab^, 28.18 ± 5.4%). We further transfected primary myocyte cultures with several more ALS-linked mutations or aberrant toxic proteins as follows: C9orf72-PR_50_, C9orf72-GR_50_ (Wen et al., 2014), and TDP43^A315T^ and used empty-GFP vector as a control. Transfected myocytes exhibit normal morphology and fusion in comparison with the WT muscle culture in our system (Fig. 2-2). Nevertheless, all ALS-causing mutations that we examined recapitulated the phenotypes we described previously in *SOD1^G93A^* (Fig. 2*F*; GFP, 40.65 ± 16%; GR_50_, 5.2 ± 3.49%; PR_50_, 0 ± 0%; TDP43^A315T^, 8.75 ± 6.39%). These results suggest that the dysregulated secretion of factors from ALS mutant muscles takes place, which in turn activates axon retraction and degeneration. Because muscles can either secrete positive or negative signaling molecules, we could not determine whether our observation within this assay originates from an increase in the release of destabilizing factors or the diminished release of positive factors. To this end, we collected muscle-CM from WT and *SOD1^G93A^* muscle cultures in complete medium containing positive factors, such as BDNF and GDNF, as was previously performed in mass culture (Nagai et al., 2007), and ultimately applied it only to the distal axons of both WT and *SOD1^G93A^* MNs (Fig. 2*G*,*H*). Interestingly, we observed that axon degeneration occurs only when *SOD1^G93A^* myocyte-CM is applied to *SOD1^G93A^* axons (Fig. 2*H*; LM, 3.72 ± 1.15%; *SOD1^G93A^*, 34.7 ± 4%), whereas in all other combinations the axons remained intact (Fig. 2-3). To further determine whether Type 3 Semaphorins, such as Sema3A, contribute to MN axon degeneration in this assay, we investigated whether NRP1-blocking antibody application can block this phenotype. Here again, we observed a rescue effect by this treatment (Fig. 2*H*; *SOD1^G93A^* + NRP1-antibody 18.6 ± 7%), although the protection was incomplete. These results reinforce our hypothesis, suggesting that ALS-mutated muscles secrete destabilizing factors, such as Sema3A. Importantly, these results emphasize that *SOD1^G93A^* MNs exhibit a higher sensitivity to degeneration, and support the MN unique vulnerability as well as the non–cell-autonomous mechanism of ALS. Interestingly, previous attempts to block Sema3A signaling in *SOD1^G93A^* mice using either a similar antibody approach or by crossing transgenic mice expressing a truncated form of Sema3A with *SOD1^G93A^* mice also resulted in only a mild improvement or none at all of motor functions (Venkova et al., 2014; Moloney et al., 2017). This suggests that Sema3A plays a complex role in MNs and that perhaps other related proteins are involved. This also led us to investigate whether a wider deregulation of secreted factors released by the diseased muscles exists. Indeed, examining other members of the Semaphorin family, we found that the percentage of NMJs expressing Sema3B, as well as NRP2 is elevated in the *SOD1^G93A^* ALS model (Fig. 2-4). Therefore, we concluded that the destabilizing effect of ALS muscles over MN axons involves more than a single factor; thus, it cannot be blocked or rescued by targeting one factor at a time. Moreover, the multiplicity of effectors suggests that a higher-order regulator, such as miRNA, might be involved in this process.

Movie 3. HB9::GFP axonal growth toward *SOD1^G93A^* myocytes. Time-lapse image series of HB9::GFP axons in the microgroove compartment of an MFC with *SOD1^G93A^* myocytes in the distal compartment (top). Scale bar, 50 μm. 10.1523/JNEUROSCI.3037-17.2018.video.3

Movie 4. HB9::GFP axonal growth toward LM myocytes. Time-lapse image series of HB9::GFP axons in the microgroove compartment of an MFC with LM myocytes in the distal compartment (top). Scale bar, 50 μm. 10.1523/JNEUROSCI.3037-17.2018.video.4

### miR126-5p is downregulated in ALS models and modulates Sema3A, Sema3B, NRP1, and NRP2 protein expression levels

To identify the mechanism underlying the elevated levels of various secreted destabilizing factors in muscles of ALS models, we scanned for alterations in miRNAs (mIRs) that can regulate the expression of multiple proteins. miRs have been previously linked to MN toxicity in ALS (Haramati et al., 2010). We used Nanostring miRNA-chip technology to screen for alterations in ∼800 miRs of presymptomatic P60 *SOD1^G93A^* mice and their LM controls. The screen yielded 8 significantly altered miRs (Fig. 3*A*; Fig. 3-1). Because we found that Sema3A levels were elevated in muscles, we narrowed our focus to those miRs that were reduced and that could regulate its expression, specifically miR126-5p and miR133a (Fig. 3*B*; the mean fold change over LM: *SOD1^G93A^*, 0.74 ± 0.03; LM, 1 ± 0.03; Fig. 3-1). A targeted search for these miRs in databases (miRDB, Pictar, miRbase, and miRTarBase) revealed that both miRs are predicted to regulate Semaphorin signaling genes as well as other relevant transcripts of ALS-related genes. Interestingly, we previously described deep-sequencing analyses of primary MN cultures expressing *SOD1^G93A^* or TDP43^A315T^ and demonstrated that miR126-5p is also correspondingly decreased in diseased MN axons, but not in their soma (Rotem et al., 2017). This information led us to further focus our investigation on miR126-5p. We used qPCR to validate that miR-126–5p levels in *SOD1^G93A^* GC muscles point to a similar trend (Fig. 3*C*; the mean fold change over LM: *SOD1^G93A^*, 0.47 ± 0.2; LM, 1 ± 0.45). To verify that miR126-5p can regulate the expression of Semaphorin3 and Neuropilin signaling members, we transfected HeLa cells, which are known to endogenously express Sema3A, Sema3B, NRP1, and NRP2 (Fujita et al., 2001), with miR126-5p or with the irrelevant miR142, which is not predicted to target any of these genes, as a negative control. To this end, we isolated RNA from these cultures and performed qPCR analysis to determine the mRNA levels of Sema3A, Sema3B, NRP1, and NRP2 (Fig. 3*D–G*). Our results indicate that miR126-5p specifically targets Sema3A, NRP1, Sema3B, and NRP2 (RT-PCR: mean ΔCt-NRP1: miR126, 3.79 ± 0.71; miR142, 2.83 ± 0.57; ΔCt-Sema3A: miR126, 4.84 ± 0.22; miR142, 3.84 ± 0.34; ΔCt-NRP2: miR126, 7.6 ± 0.30; miR142, 6.2 ± 0.37; ΔCt-Sema3B: miR126, 8.1 ± 0.10; miR142, 7.05 ± 0.14). To investigate whether miR126-5p overexpression can also inhibit Sema3A function, we used a recently described impedance-based approach. U87MG human glioblastoma cells, which express NRP1 (Fig. 3-2*A*) and were used previously specifically in this assay (Birger et al., 2015), were transfected to overexpress miR126-5p or miR142 as a control. One day after transfection, cells were resuspended and plated in xCELLigence multiwell electric plates. The next day, Sema3A was added to the cultures, and any morphological or adhesive changes were monitored by the impedance readout. As demonstrated by TIRF imaging (Fig. 3*H*), adding Sema3A to responsive cells, such as U87MG cells, induces their detachment from the culture dish. This detachment can be measured as a decrease in impedance (Fig. 3-2*B*). Shortly after Sema3A was added to the cultures, cells expressing miR142 exhibited decreased impedance, whereas cells expressing miR126-5p did not respond to Sema3A in the medium and kept growing with a corresponding increase in impedance (Fig. 3*I*). Hence, we showed that the excess production of destabilizing factors in ALS is likely to be mediated downstream of a deregulation in miR126-5p.

10.1523/JNEUROSCI.3037-17.2018.f3-1 Figure 3-1 **NanoString miR chip screen reveals the most significantly altered miRs in the GC muscles of SOD1^G93A^ compared with LM mice.** List of the most significantly altered miRs detected in the muscles of P60 SOD1^G93A^ mice compared to their LM. Data were normalized to the 100 most abundant miRs in the tissue; *p<0.05 (Student’s t-test; n=3). Only two miRs were significantly down-regulated in SOD1^G93A^ muscles: miR126-5p and miR133a; **p<0.01 (Student’s t-test; n=3). Download Figure 3-1, TIF file

10.1523/JNEUROSCI.3037-17.2018.f3-2 Figure 3-2 **U87MG impedance measurement after Sema3A application.** (A) Western blot analysis of the U87MG cell line after 3 days in culture reveals the presence of NRP1 protein (∼120kDa). The expression of NRP1 protein did not change after m.Cherry transfection. ERK (∼42-44 kDa) was used as a loading control. (B) Impedance recording of live U87 cells over time shows the responsiveness to Sema3A within minutes after its application, unlike the cells that were treated with the control medium. Download Figure 3-2, TIF file

### Overexpression of miR126-5p can block *SOD1^G93A^* muscle toxicity

We overexpressed miR126-5p in SOD^G93A^ myocyte cultures and quantified Sema3A levels in their culture extract as well as in their CM. Western blot analysis indicated that Sema3A levels in both the culture extract and CM are depleted, compared with miR142 (Fig. 4*A*,*B*; the mean fold change over SOD1^miR142^: Muscle extract-SOD1^miR126^, 0.24 ± 0.1; SOD1^miR142^, 1 ± 0.4 Muscle-CM-SOD1^miR126^, 0.63 ± 0.03; SOD1^miR142^, 1 ± 0.13). Next, we investigated whether overexpressing miR126-5p in both *SOD1^G93A^* and PR_50_ myocytes can rescue the negative effect on MN growth that we observed before. To this end, primary myoblasts were transfected to overexpress either miR126-5p (SOD1^miR126^; PR_50_^miR126^) or miR142 (SOD1^miR142^; PR_50_^miR142^) and were then plated in the distal compartment of the MFC. Myoblasts were differentiated into mature myocytes while expressing the miRs for 7 d, after which HB9::GFP explants were cultured in the proximal compartment. Once axons reached the microgrooves, their extension toward the muscle compartment was monitored for 16 h (Fig. 4*C*). Evidently, cocultures with SOD1^miR126^ and PR_50_^miR126^ myocytes retained wild-type behavior and manifested a clear rescue effect on the rate of axon traversal (Fig. 4*D*,*E*; the mean traversal rate of axons: SOD1^miR126^, 40.77 ± 6.68%; SOD1^miR142^, 12 ± 7.6%; PR_50_^miR126^, 45.6 ± 9.4%; PR50^miR142^, 16 ± 3.6%). Thus far, we have shown that myocytes expressing various ALS-linked mutations facilitate MN axon degeneration and delay their growth in a simplified compartmental coculture assay. However, while observing the cocultures for longer periods, we found that axons eventually do traverse the muscle compartment and form functional synapses with the myocytes. Using an image-based method that we recently developed to quantify contraction and assess the innervation in *in vitro* cocultures (Ionescu et al., 2016; Zahavi et al., 2017), we observed that the contractile behavior of innervated *SOD1^G93A^* myocytes is significantly different from that of innervated LM myocytes, which tend to contract in a bursting pattern (Fig. 4*F*,*G*; Movies 5, 6). Whereas 37% of innervated myocytes contract in a bursting pattern, only 18% of the innervated *SOD1^G93A^* myocytes contract in this pattern (the mean rate of bursting innervated myocytes: LM, 37.23 ± 2.8%; *SOD1^G93A^*, 18.5 ± 2.03%). Strikingly, SOD1^miR126^ myocytes retain the same rate of bursting myocytes as the LM myocytes (Fig. 4*G*; the mean rate of bursting innervated myocytes: SOD1^miR126^, 37.66 ± 4.29%; SOD1^miR142^, 26.26 ± 0.59%). Hence, miR126-5p is an effective regulator of muscle-secreted factors, such as Sema3, and can rescue the detrimental effect of destabilizing factors on MN axons, as well as on NMJ function and maintenance *in vitro*.

Movie 5. Myocyte exhibiting bursting contracted behavior. Time-lapse image series of innervated SOD^G93A^ expressing miR126-5p myocyte exhibiting bursting contracted behavior. Scale bar, 25 μm. 10.1523/JNEUROSCI.3037-17.2018.video.5

Movie 6. Myocyte exhibiting nonbursting contracted behavior. Time-lapse image series of innervated SOD^G93A^ expressing miR142 myocyte exhibiting nonbursting behavior. Scale bar, 25 μm. 10.1523/JNEUROSCI.3037-17.2018.video.6

### miR126-5p transiently rescues early motor phenotypes of *SOD1^G93A^* mice *in vivo*

**Figure 5.**
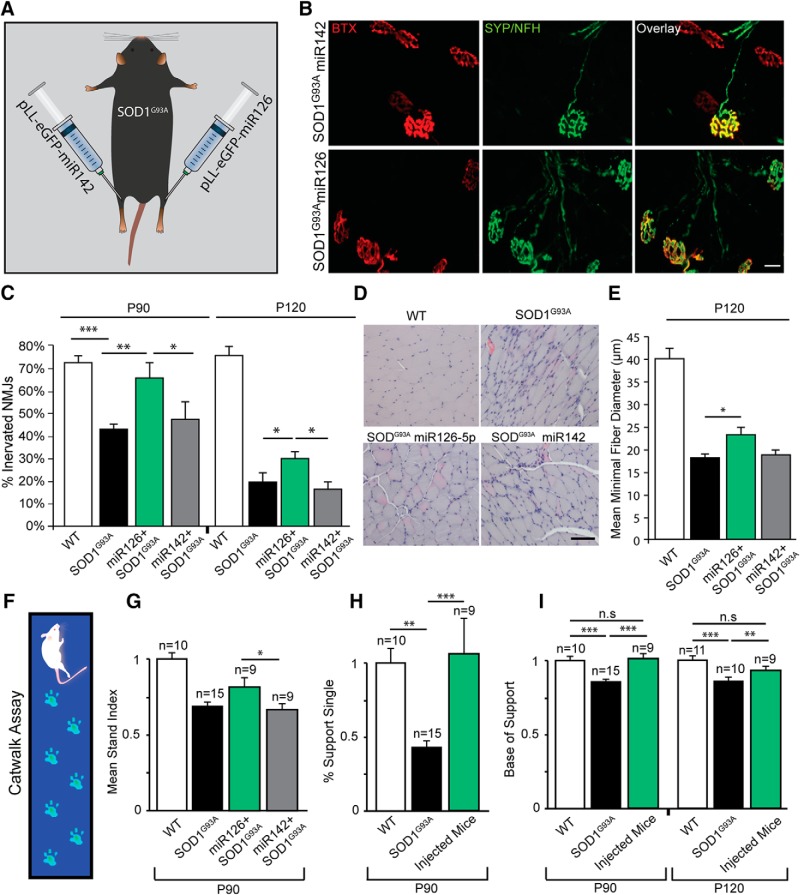
pLL-eGFP-miR126-5p injected into GC muscles of presymptomatic *SOD1^G93A^* mice transiently rescues the early phenotype appearance *in vivo*. ***A***, Schematic view of the *in vivo* experimental procedure. *SOD1^G93A^* mice were injected with either pLL-eGFP-miR126-5p or pLL-eGFP-miR142 in their right or left GC muscles, respectively. Viral infection was validated (Figure 5-1). ***B***, Representative whole-mount NMJ immunostaining of ∼P90 *SOD1^G93A^* GC muscles injected with either miR126-5p or miR142 lenti vectors. Red represents BTX. Green represents NFH + synaptophysin in presynaptic neurons. Scale bar, 20 μm. ***C***, The percentage of innervated NMJs in miR126-5p-injected muscles is higher compared with its controls in both P90 and P120 (P90: Student's *t* test, *n* = 6, *p = 0.0475, **p = 0.001245; P120: Student's *t* test, *n* = 5, *p = 0.043, **p = 0.0096). ***D***, Representative histological images of P120 WT, *SOD1^G93A^*, miR126-5p, and miR142 GC muscle cross sections after H&E staining. Scale bar, 100 μm. ***E***, Semiquantification of a GC cross section from ***D*** shows a significant increase in the minimal muscle fiber diameter of muscles that were injected with miR126-5p (P120: Student's *t* test, *n* = 4, *p = 0.031). ***F***, Illustration of the CatWalk XT gait analysis system that monitors mouse footprints. ***G***, Gait analysis MSI parameter indicates the speed at which the paw loses contact with the surface. The MSI for the P90 miR126-5p-injected limbs was significantly higher than for miR142-injected limbs (Student's *t* test, *p = 0.0355). ***H***, Gait analysis percentage single-support parameter indicates the relative duration of contact of a single paw on the glass floor. The percentage in which the injected animals were used along the run with a single paw was significantly higher compared with *SOD1^G93A^* mice and showed similarity to the WT control (Student's *t* test, *SOD1^G93A^*-injected, ***p = 0.0004; WT-*SOD1^G93A^*, ***p = 0.000003). ***I***, Gait analysis base of support parameter indicates the average width between the hindpaws. The base of support of both P90- and P120-injected mice was significant higher compared with *SOD1^G93A^* (Student's *t* test, P90 *SOD1^G93A^*-injected, ***p = 0.0000006; WT-*SOD1^G93A^*, ***p = 0.000007; P120 *SOD1^G93A^*-injected, ***p = 0. 0.00003; WT-*SOD1^G93A^*, ***p = 0.000000009).

NMJ disruption, muscle morphology abnormalities, and hindlimb misprints are major phenotypes in *SOD1^G93A^* mice (Gurney et al., 1994). To determine whether miR126-5p can moderate those phenotypes, we injected *SOD1^G93A^* mice with either pLL-eGFP-miR126 (SOD1^miR126^) or pLL-eGFP-miR142 (SOD1^miR142^) into the right and left GC muscles of presymptomatic mice (P60), respectively (Fig. 5*A*). Virus expression was validated both *in vitro* on MNs and in muscle cultures (Fig. 5-1*A*) as well as *in vivo* at the transcript and protein levels (Fig. 5-1*B*,*C*). Importantly, we observed a decrease in the number of NMJs expressing Sema3A in the pLL-eGFP-miR126-5p-injected GC muscles in comparison with the miR142 group, suggesting that miR-126–5p is active in the injected tissue (Fig. 5-1*D*). Next, we performed a series of histological analyses, followed by motor behavioral tests at two time points after injection: at the age at which mice typically begin exhibiting ALS phenotypes (P90) as well as in the disease end stage (P120) (Fischer et al., 2004). Because NMJ disruption is a key process in ALS, we sought to determine whether overexpression of miR126-5p results in reduced NMJ disruption. Briefly, both the left and right GC muscles were fixed and stained for synaptic markers of the NMJ (Fig. 5*B*). Quantifying the percentage of intact NMJs at P90 injected mice revealed a significantly higher innervation rate in miR126-5p-expressing muscles compared with both mock-treated and *SOD1^G93A^* muscles (Fig. 5*C*; P90: WT, 71.58 ± 3.32%; SOD^G93A^, 42.58 ± 2.64%; SOD1^miR126^, 64.25 ± 5.8%; SOD1^miR142^, 46.54 ± 7.2%). Furthermore, careful analysis at P120 also identified a mild rescue by miR-126–5p overexpression. (Fig. 5*C*; P120: WT, 74.35 ± 4.74%; SOD^G93A^, 20.12 ± 5.01%; SOD1^miR126^, 30.82 ± 3.97%; SOD1^miR142^, 18.18 ± 3.12%). Next, we performed histological analyses to determine muscle fiber wasting and tissue abnormalities (Fig. 5*D*,*E*). P120 GC muscles of WT, *SOD1^G93A^*, and both SOD1^miR126^ and SOD1^miR142^ were stained with H&E for histological examination, and the minimal diameter of myofibers was analyzed as described in Materials and Methods. We observed a mild, but significant, increase in the minimal fiber size of the SOD1^miR126^-injected muscle compared with the SOD1^miR142^ mock control (Fig. 5*C*; P120: WT, 40.25 ± 2.28%; SOD^G93A^, 18.5 ± 0.64%; SOD1^miR126^, 23 ± 1.87; SOD1^miR142^, 19 ± 1.47). Last, we performed a behavioral test using the CatWalk gait analysis technique. This video-based method is a computerized version of the ink bath assay and provides an objective and dynamic wide range of gait analyses (Deumens et al., 2007). Moreover, it has been used before specifically with the *SOD1^G93A^* mouse model and displayed significant differences in several parameters (Mead et al., 2011; Gerber et al., 2012) (Fig. 5*F*). One output is the Mean Stand Index (MSI), which measures the speed at which the paws detach from the walking surface. Because aged *SOD1^G93A^* mice suffer motor defects, their MSI values for both hindlimbs are dramatically lower than their LM values. Remarkably, the MSI values of the SOD1^miR126^-injected limbs were significantly higher at P90 and similar to the LM control values, whereas the SOD1^miR142^-injected limb was reminiscent of typical *SOD1^G93A^* behavior (Fig. 5*G*; mean fold change over WT: *SOD1^G93A^*, 0.68 ± 0.02; SOD1^miR126^, 0.74 ± 0.06; SOD1^miR142^, 0.65 ± 0.04; LM, 1 ± 0.04). We also examined other established parameters that have been shown to be altered in the SOD^G93A^ model (Mead et al., 2011). We specifically focused on the percentage of single-support parameter, which indicates the relative duration of contact of all combined paws with the glass floor, and on the base of support parameter, which indicates the average width of limb spreading between both front, or both hindpaws. Remarkably, we observed a significant rescue phenotype for both parameters in the injected mice at age of P90. Furthermore, the improvement in base of support parameter persisted also in P120 (Fig. 5*H*,*I*; Percentage of Support Single: *SOD1^G93A^*, 0.43 ± 0.05; injected, 1.06 ± 0.24; LM, 1 ± 0.1; Base of Support, P90: *SOD1^G93A^*, 0.87 ± 0.01; injected, 1.02 ± 0.02; LM, 1 ± 0.02; P120: *SOD1^G93A^*, 0.858 ± 0.02; injected, 0.95 ± 0.02; LM, 1 ± 0.02).

10.1523/JNEUROSCI.3037-17.2018.f5-1 Figure 5-1 **Validation of pLL-eGFP-miR virus expression *in vitro & in vivo.***(A) Representative images of *in vitro* pLL-eGFP-miR-126-5p infection of both MNs culture (left panel) and muscle culture (right panel) demonstrate the pLL-eGFP-miR construct’s ability to infect and be expressed specifically in those tissues. Titer: 6x10^9^ Scale Bar: 100um (B) Western blot analysis of GFP protein levels of pLL-eGFP-miR126-5p and pLL-eGFP-miR142-injected GC muscle extract compared to non-injected muscle tissues reveals an increase in the GFP protein level only in muscles that were injected with either pLL-eGFP-miR126-5p or pLL-eGFP-miR142 viruses. Transfected myocyte cultures overexpressing GFP were used as a positive control. (C) qPCR analysis of GFP mRNA levels in pLL-eGFP-miR126-5p and pLL-eGFP-miR142-injected GC muscle extract compared to non-injected muscle tissues reveals an increase in GFP transcripts only in muscles that were injected with either pLL-eGFP-miR126-5p or pLL-eGFP-miR142 viruses (the mean fold change over non-injection: +injection 6.98 ± 2.38; -injection 1; n=4; n=1, respectively). (D-E) Whole-muscle NMJ immunostaining of injected SOD1^G93A^ muscles with pLL-eGFP-miR126 or pLL-eGFP-miR142. Red indicates postsynaptic acetylcholine receptors labeled with BTX, gray indicates Sema3A. The percentage of NMJs expressing Sema3A in pLL-eGFP-miR126-injected muscles is lower compared with the pLL-eGFP-miR142-injected muscle control (The mean percentage of NMJs expressing Sema3A: miR126-5p 16%± 1%; injection of miR142 26% ± 5%; Student’s t-test, n=5, * p=0.03357). Download Figure 5-1, TIF file

Together, we demonstrated *in vivo* that miR126-5p reduces the detrimental effects of muscle-secreted destabilizing factors, such as Sema3A, on MN axons and motor function in ALS models.

## Discussion

**Figure 6.**
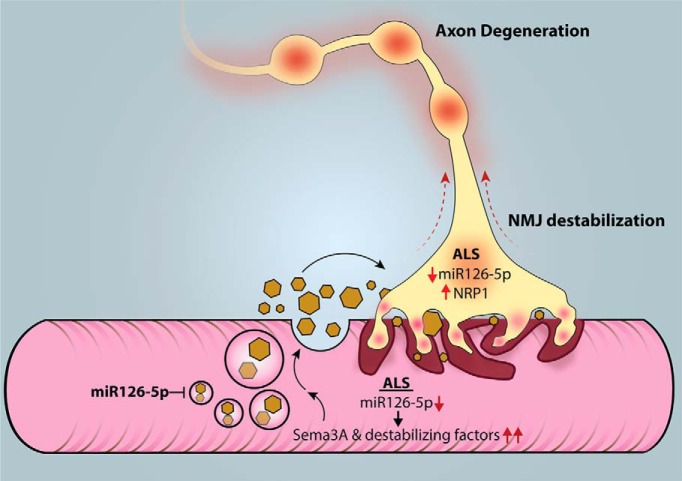
Alterations in Semaphorin3A regulation by miR126-5p trigger MN degeneration in ALS. miR126-5p is a negative regulator of Sema3 signaling in skeletal muscles. Downregulation of miR126-5p in ALS disease drives the overexpression and secretion of Sema3A and potentially other NMJ-destabilizing factors in skeletal muscles. The downregulation in miR126-5p in diseased MNs drives the overexpression of NRP1 specifically in axons. The excess binding and activation of the NRP1 receptor by its overexpressed ligand Sema3A as a result of miR126-5p alteration promote NMJ disruption and axon degeneration in a spatially confined process.

In this work, we demonstrated that the muscle toxicity in ALS is mediated by miR126-5p. We provided one specific mechanism for a well-described molecule (Sema3A), by which miR126-5p contributes to ALS pathology. We have also demonstrated that miR126-5p alterations facilitate axon degeneration and NMJ disruption in an ALS model as an outcome of presymptomatic elevations in the production and secretion of their target genes, which encode for destabilizing factors, such as Sema3 family members. Overexpressing miR126-5p in *SOD1^G93A^* muscles inhibits the neurodegenerative process. These findings reveal how alterations in miR126-5p can be toxic to MNs, and identify a non–cell-autonomous neurodegeneration process in ALS (Fig. 6).

### miR126-5p as a master regulator of proper NMJ function

Our results indicate that the expression of ALS-causative mutations results in the secretion of repellent factors, including a number of Type 3 Semaphorins and potentially other factors. It is thus likely that a general gene repression mechanism, specifically miR system, is altered under such conditions. This assumption is also consistent with the fact that miR alterations are apparent in various neurodegenerative diseases, including ALS (Haramati et al., 2010). Here we identified such an miR and showed how alterations in this specific miR can regulate the essential signaling pathways in MNs and can trigger neurodegeneration. Intriguingly, and in line with our findings, a very recent paper demonstrated a mechanism by which miR126-5p modulates Sema3A expression through SetD5 expression, and it emphasizes its positive effect on retinal endothelial cells' survival (Villain et al., 2018). However, aside from targeting Sema3A and Sema3B, as well as NRP1 and NRP2, miR126-5p is predicted to regulate other Semaphorin signaling factors, such as Sema6D, PLXNA2, JNK2, JNK3, and PTEN. In addition, miR126-5p can regulate the ALS and motor-unit-related genes VEGF-A, SPAST, MMPs (Kaplan et al., 2014), AGRIN (Vilmont et al., 2016), and C9orf72, which are directly involved in ALS. Therefore, miR126-5p can serve as a master regulator of NMJ health by controlling multiple signaling pathways.

### Sema3 alterations in ALS: settling the contradictory reports

A critical initiating event for the mechanism outlined above is the alterations in Sema3 signaling in ALS models and patients. Sema3 family members were previously found to be upregulated in terminal Schwann cells in the NMJs of *SOD1^G93A^* mice (De Winter et al., 2006). Recently, Sema3A was also shown to be elevated in the motor cortex of postmortem ALS patients but not in their SC (Körner et al., 2016). Consistent with this, NRP1 antibodies, blocking the obligatory binding receptor for Sema3A, were injected into *SOD1^G93A^* mice as a potential treatment (Venkova et al., 2014). However, anti-NRP1 blocking antibody had only a modest effect. Moreover, a recent study demonstrated that crossing mice expressing a truncated form of Sema3A with *SOD1^G93A^* mice did not result in any rescue effect (Moloney et al., 2017). An explanation for a minor effect or not at all, as a result of blocking Sema3A activity, could be based on the idea of multiple toxic factors that play a role in ALS pathology. Another explanation for this contradiction could be the fact that Sema3A plays a more complex role in the biology of MNs. Indeed, Sema3A was shown to increase survival when added to mass cultures of mouse MNs (Molofsky et al., 2014) and human MNs (Birger et al., 2018). Consistent with this, deletion of the Sema3A gene specifically in spinal astrocytes resulted in a gradual loss of spinal MNs (Molofsky et al., 2014), thus suggesting that Sema3A has a positive effect when introduced near the cell body. When these findings are together with our results, apparently Sema3A has both positive and negative effects on MNs, perhaps depending on its specific subcellular localization. When Sema3A is secreted from muscles and targets distal axons at NMJs, it mediates their destabilization; however, when it is secreted by spinal astrocytes and targets MN soma, it acts as a survival factor. Thus, it is perhaps not surprising that a genetic approach to inhibit Sema3A in all cells in a mouse model of ALS had no effect in inhibiting the symptoms. The injection of anti-NRP1 may have been a bit more beneficial possibly either because it was able to inhibit Sema3A outside the CNS more effectively, or alternatively, because NRP1 blocks other Type 3 Semaphorins as well.

### Autonomous versus non–cell-autonomous contributions to ALS progression

ALS is considered a complex disease, with unique MN features as well as non–cell-autonomous contributions (Ilieva et al., 2009; Musarò, 2013). Some evidence suggests that the NMJ is the first compartment to be disrupted in ALS rather than the MN soma; the disease is recognized as distal axonopathy in a non–cell-autonomous process (Fischer et al., 2004; Moloney et al., 2014). Two main cell populations that have been shown to play a role in distal axonopathy are glia and muscle cells, which secrete factors that influence MN survival and health (Moloney et al., 2014; Tsitkanou et al., 2016). However, the complexity of the disease and the involvement of several tissues raise controversies regarding the contribution of each tissue to the disease pathology. With skeletal muscle, few works have concluded that muscles do not play a role in ALS pathology. Reducing hSOD^G93A^ levels by injecting siRNA against its transcript directly into muscles of the SOD^G93A^ mouse model, as well as crossing Lox SOD^G37R^ with the Cre coding sequence under the control of the muscle creatine kinase promoter, or performing manipulations using Follistatin did not affect the disease onset and survival (Miller et al., 2006). *In vitro* application of muscle-CM from SOD^G93A^-expressing muscle on healthy mass culture and ES cell-derived MNs resulted in no effect (Nagai et al., 2007). However, in contrast with these findings, evidence indicates that overexpressing mutant SOD1 protein specifically in healthy skeletal muscle induces an ALS phenotype and the degeneration of MNs, supporting a direct role for muscle in ALS physiology (Dobrowolny et al., 2008; Wong and Martin, 2010). Moreover, muscle from ALS patients and models has been shown to exhibit impaired mitochondrial function (Shi et al., 2010) and abnormalities in muscle biology (Manzano et al., 2012). Here, we demonstrated that applying presymptomatic *SOD1^G93A^* muscle-CM directly, and only on *SOD1^G93A^*-expressing MN axon tips, results in their degeneration, suggesting that both tissue types are necessary for exhibiting the disease phenotype. Furthermore, contradictory reports were published on transgenic mice expressing *SOD1^G93A^* only in the MNs. For example, Lino et al. (2002) showed a very mild phenotype, whereas Jaarsma et al. (2008) demonstrated a significant toxic effect. In this study, we showed that muscle-secreted factors are capable of modulating MN axons. Whereas wild-type muscle-secreted factors facilitate axon growth, several ALS-related mutations, expressed in muscles, result in the secretion of factors that cause retraction and degeneration when exclusively introduced to axons. At least one of these factors is Sema3A. The secretion of Sema3A by the muscle itself is likely to contribute to the instability of the MN axons. However, our results also show that ALS mutant muscles themselves cause axon degeneration and a delay in axon growth toward the muscles, but eventually the connections between axons and muscles are established. Thus, at least in our system, apparently the non–cell-autonomous contributions of the muscle are insufficient to recapitulate all the toxic effects on MNs. However, once the MNs also carry an ALS mutation, axons are more susceptible to degeneration by mutated muscle-CM (Fig. 3*G*,*H*), thus demonstrating the critical contribution of the MNs to ALS progression.

### Do diseased muscles initiate axon degeneration or inhibit regeneration?

Our data suggest that muscles are involved in modulating MN health in ALS disease. We showed, both *in vivo* and *in vitro*, that muscles secrete destabilizing factors, such as Sema3A, as well as facilitate axon degeneration and NMJ disruption. Intriguingly, a previous study demonstrated that Sema3A expression is limited only to myosin IIb positive fibers, which are prone to be disrupted first in ALS (De Winter et al., 2006). These data support our findings in which the percentage of NMJs that express Sema3A and NRP1 is reduced at the end stage of the disease, most likely along with the fast fatigue NMJs. However, although the suggested mechanism involves muscle-MN interaction, because of the nature of our experimental model, we cannot fully determine whether the mutated muscles act by initiating the degeneration of MN axons or by inhibiting their regrowth and NMJ repair, which was also suggested previously (Arbour et al., 2015). Perhaps muscle toxicity plays an active role in both pathways. However, future efforts should be made to resolve this issue.

### miRs as a potential therapeutic strategy for ALS disease

In this paper, we demonstrated a positive effect of miR126-5p on ALS disease progression and suggested a potential therapeutic strategy for ALS disease. Nevertheless, our *in vivo* data show that the most significant positive effect of miR126-5p on ALS pathology was achieved at P90, whereas at later stages only modest effects were achieved. These results point to miR126-5p as a targeted treatment for an early phenotype but without a sustained beneficial contribution at later stages of ALS disease. However, keeping in mind that we injected miR126-5p into small parts of the whole GC muscle and only once at P60, as well as the fact that the efficiency of the procedure of the injection can also affect the efficacy of this treatment, we cannot rule out the possibility that a broader test of long-term efficacy will result in a stronger and more positive outcome. An alternative future study should address this issue by crossing a conditional tissue-specific knock-out of miR126-5p mice with *SOD1^G93A^*.

Considering that ALS is a multifactorial disease, and that miRs are predicted to regulate a wide range of metabolic and signaling pathways, manipulating their subcellular levels in neurons, muscles, or glia, miRs should generally be explored as a potential therapeutic strategy or tool for treatment of ALS and possibly other neurodegenerative diseases.
