# Supplementary figures and images for: miR126-5p Downregulation Facilitates Axon Degeneration and NMJ Disruption via a Non–Cell-Autonomous Mechanism in ALS
Source: J Neurosci. 2018 Jun 13;38(24):5478–94. doi: 10.1523/JNEUROSCI.3037-17.2018 (PMC6001038; doi:10.1523/JNEUROSCI.3037-17.2018)

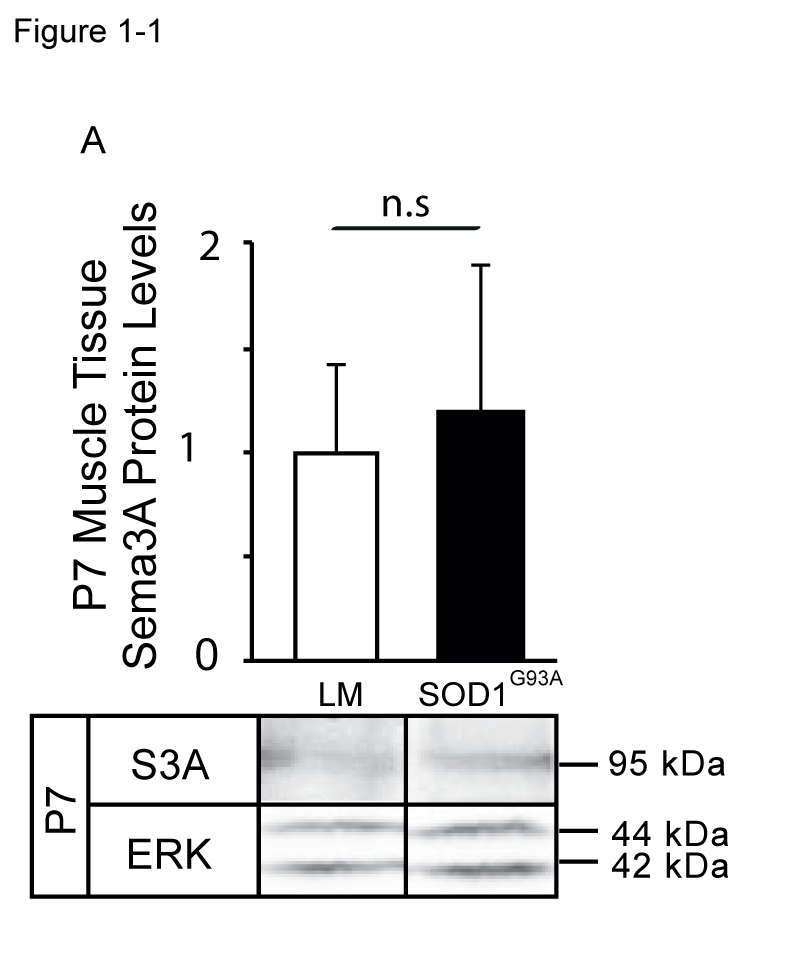

Supplement: Figure 1-1 [file zns999180838so1.tif]

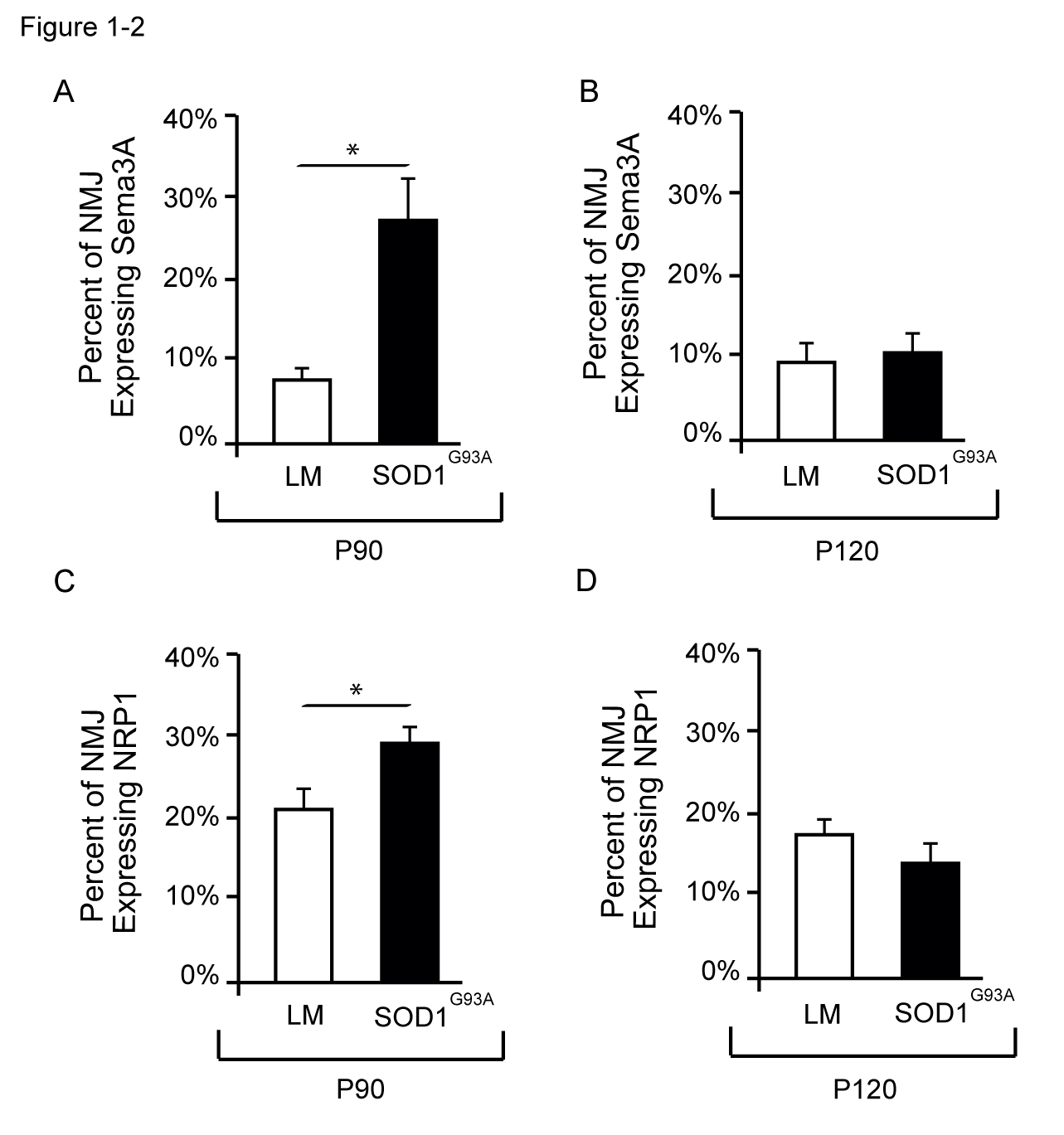

Supplement: Figure 1-2 [file zns999180838so2.tif]

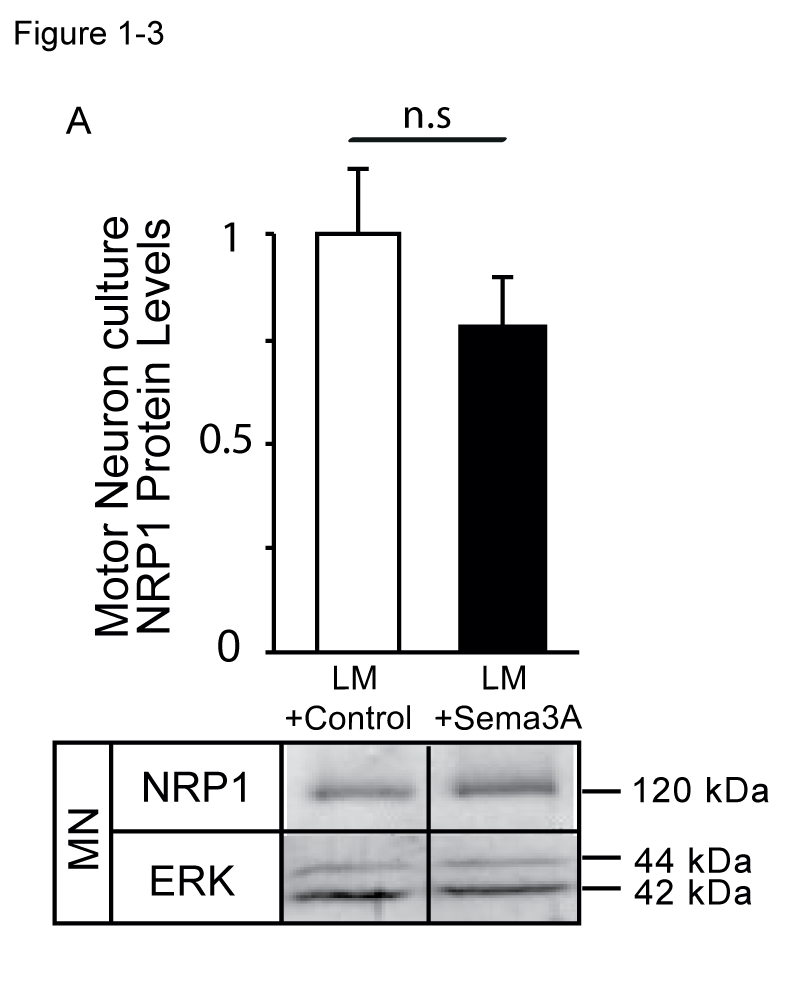

Supplement: Figure 1-3 [file zns999180838so3.tif]

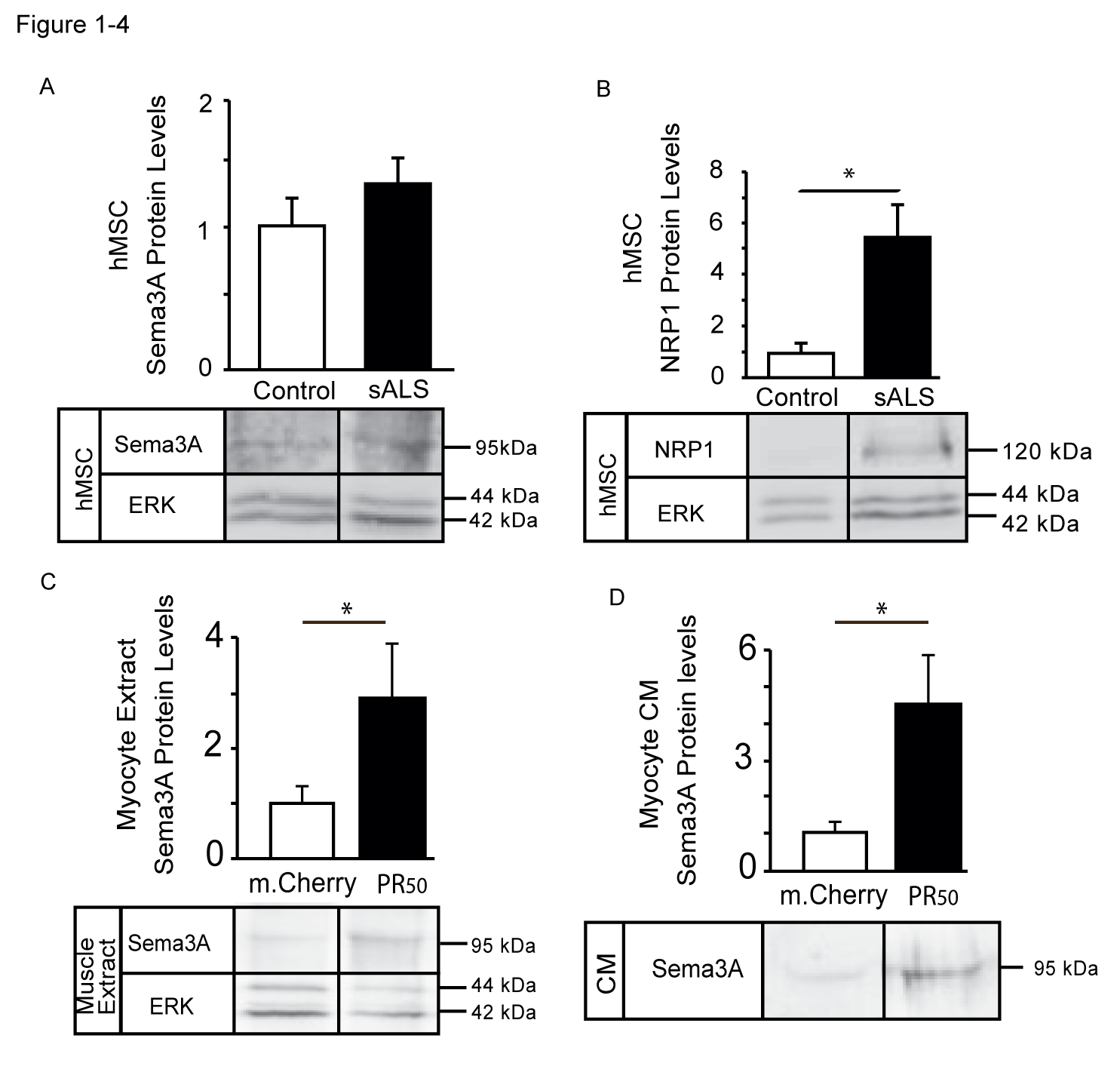

Supplement: Figure 1-4 [file zns999180838so4.tif]

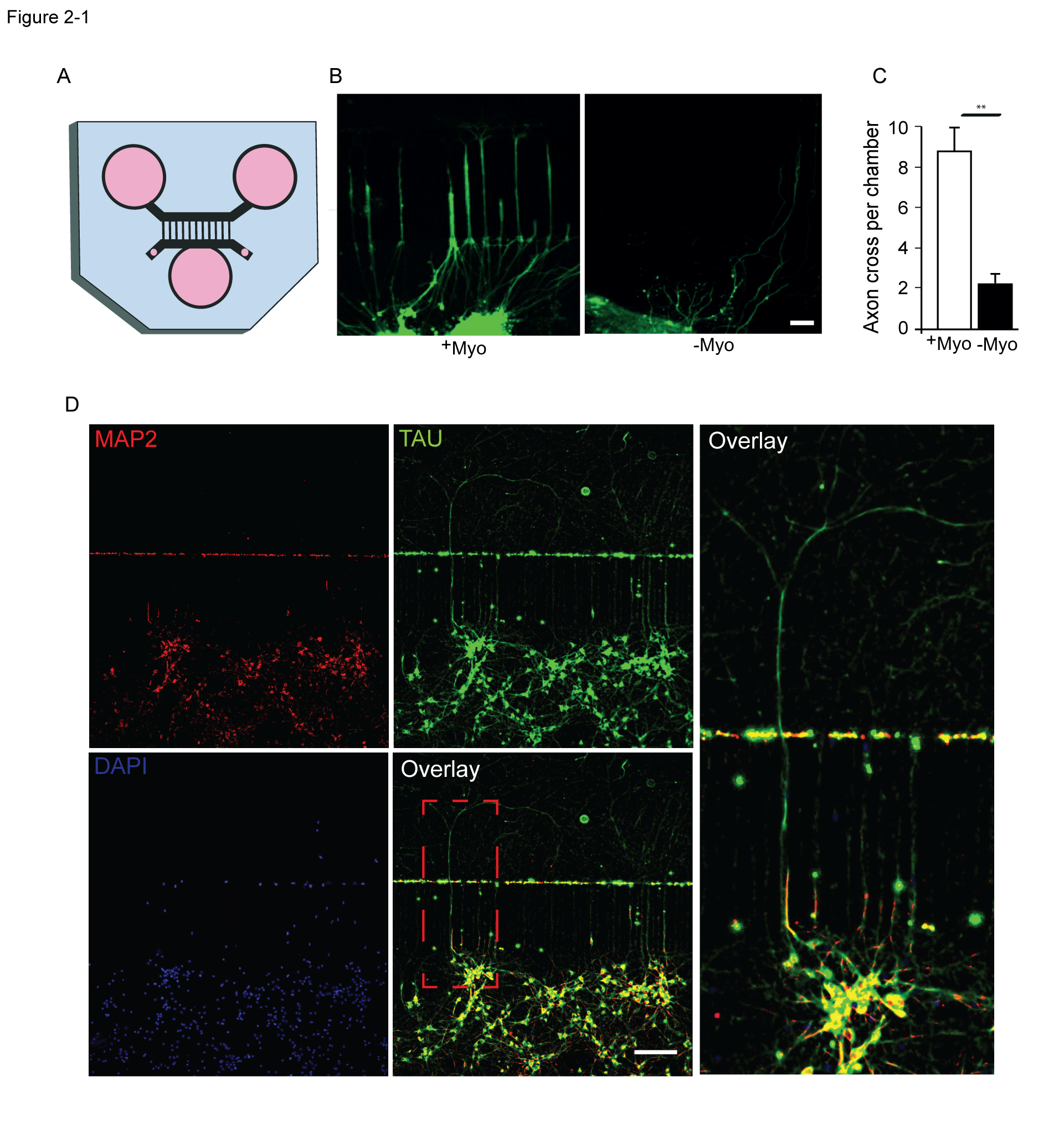

Supplement: Figure 2-1 [file zns999180838so5.tif]

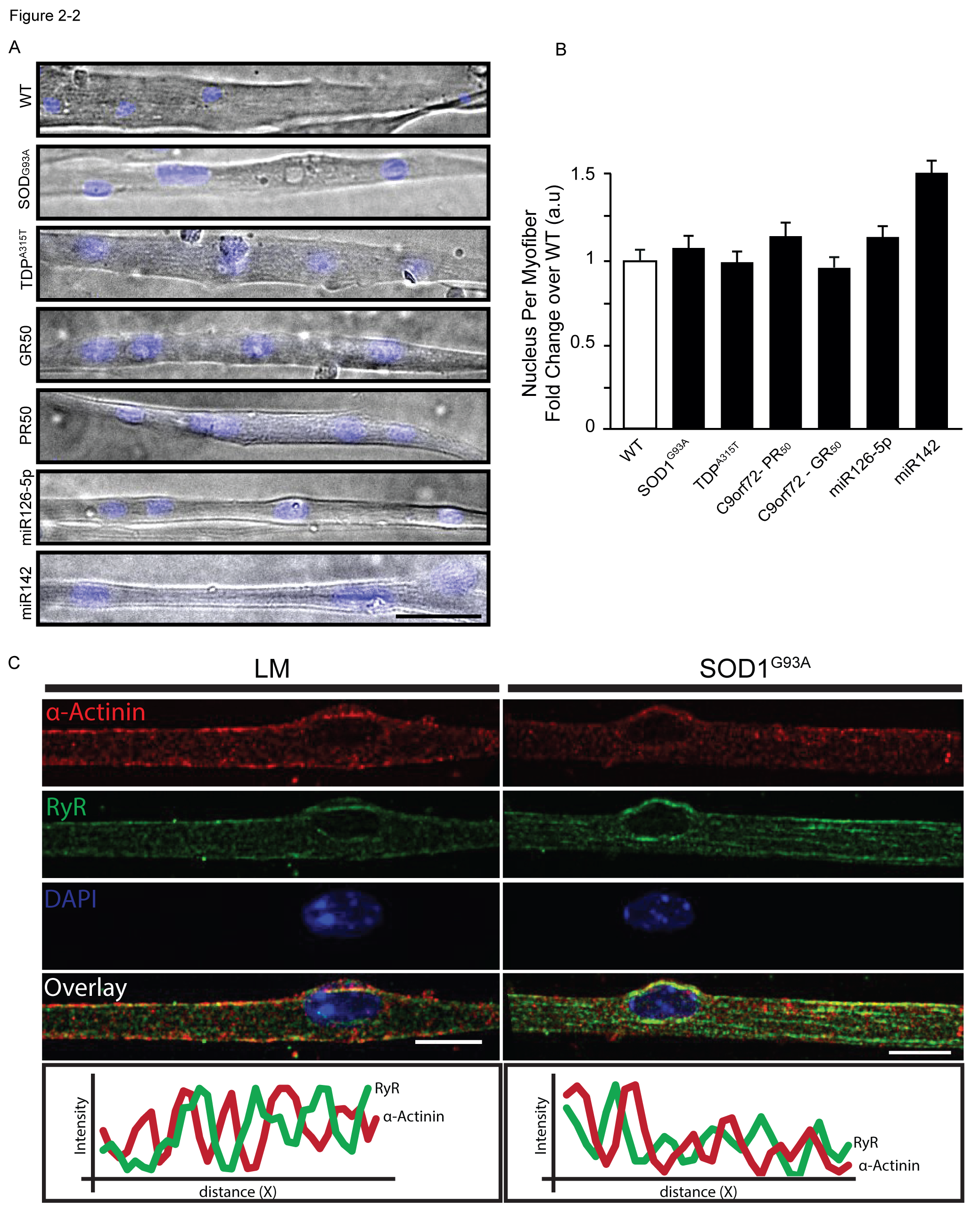

Supplement: Figure 2-2 [file zns999180838so6.tif]

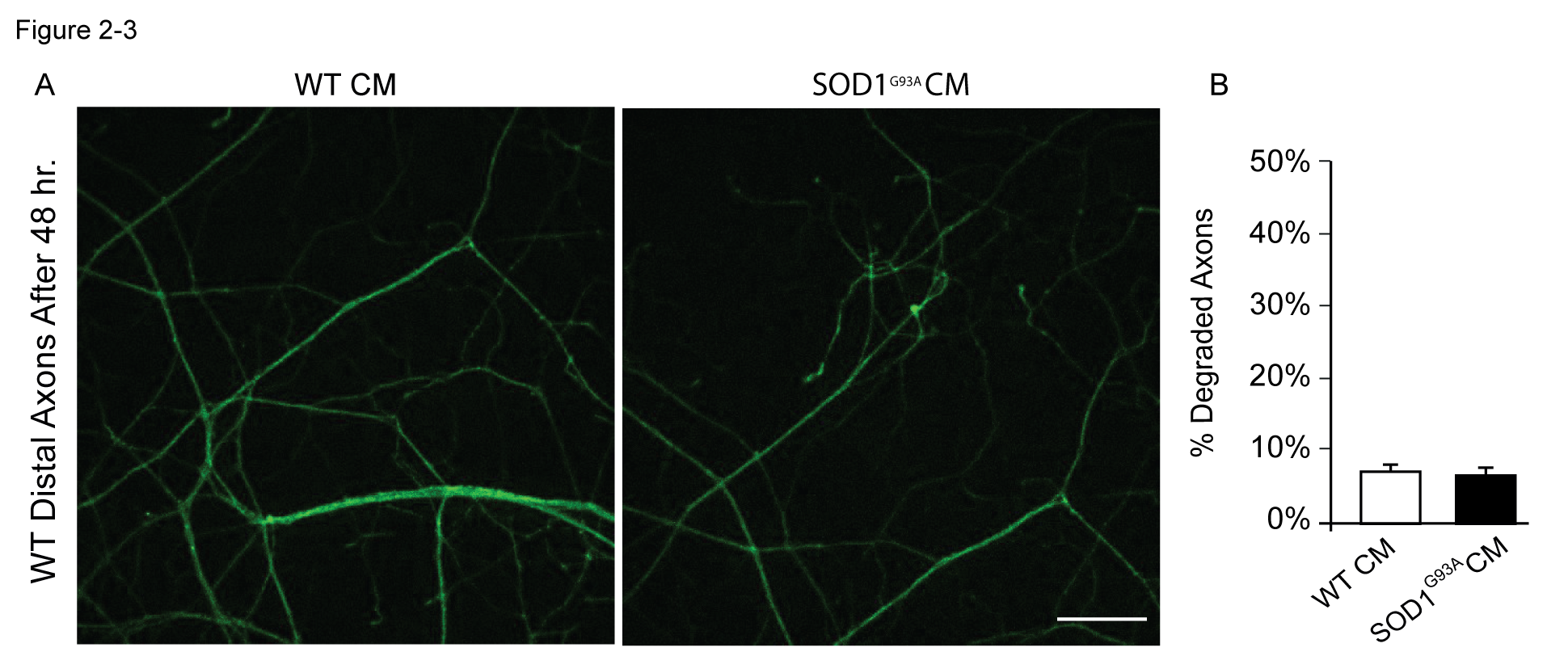

Supplement: Figure 2-3 [file zns999180838so7.tif]

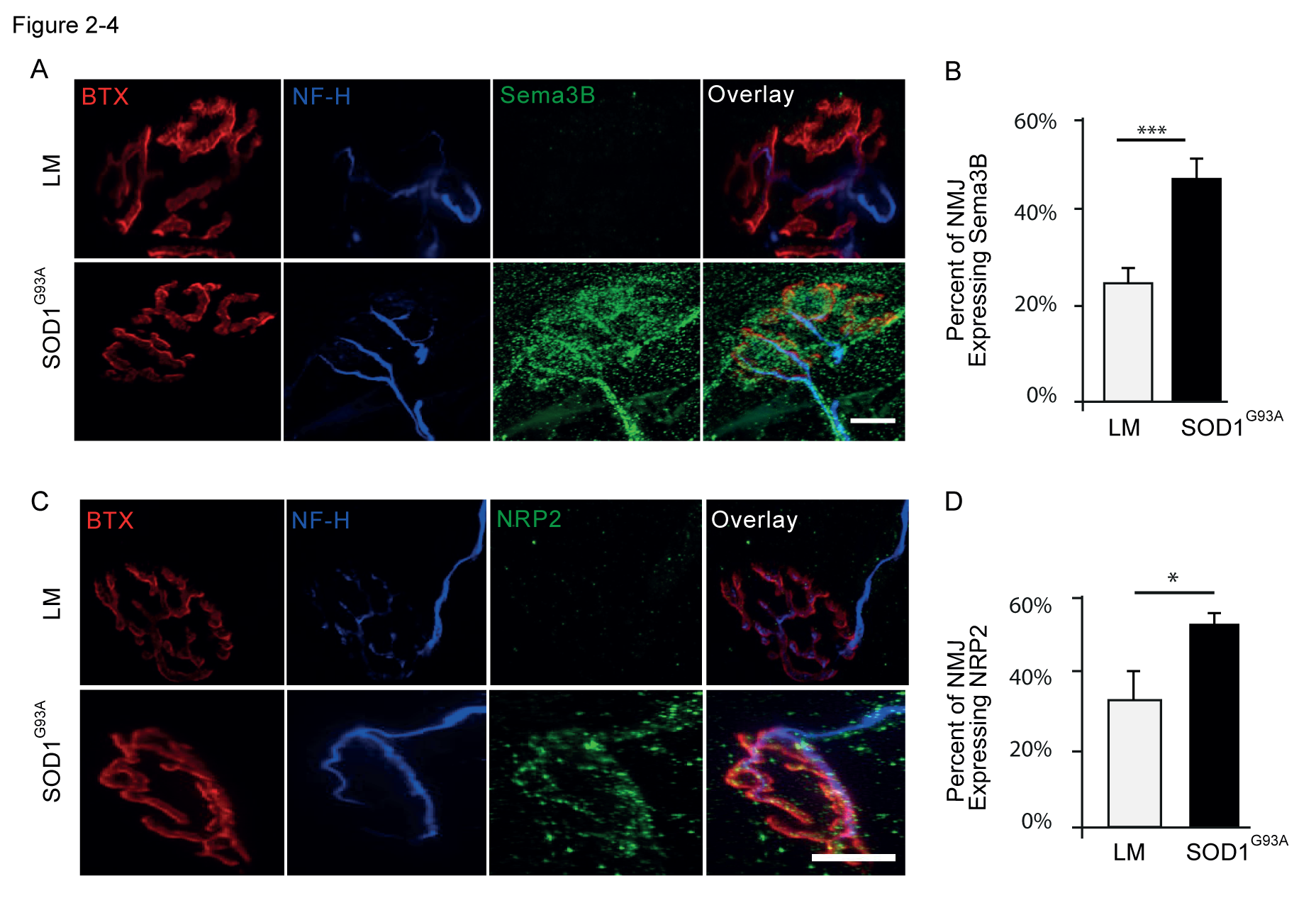

Supplement: Figure 2-4 [file zns999180838so8.tif]

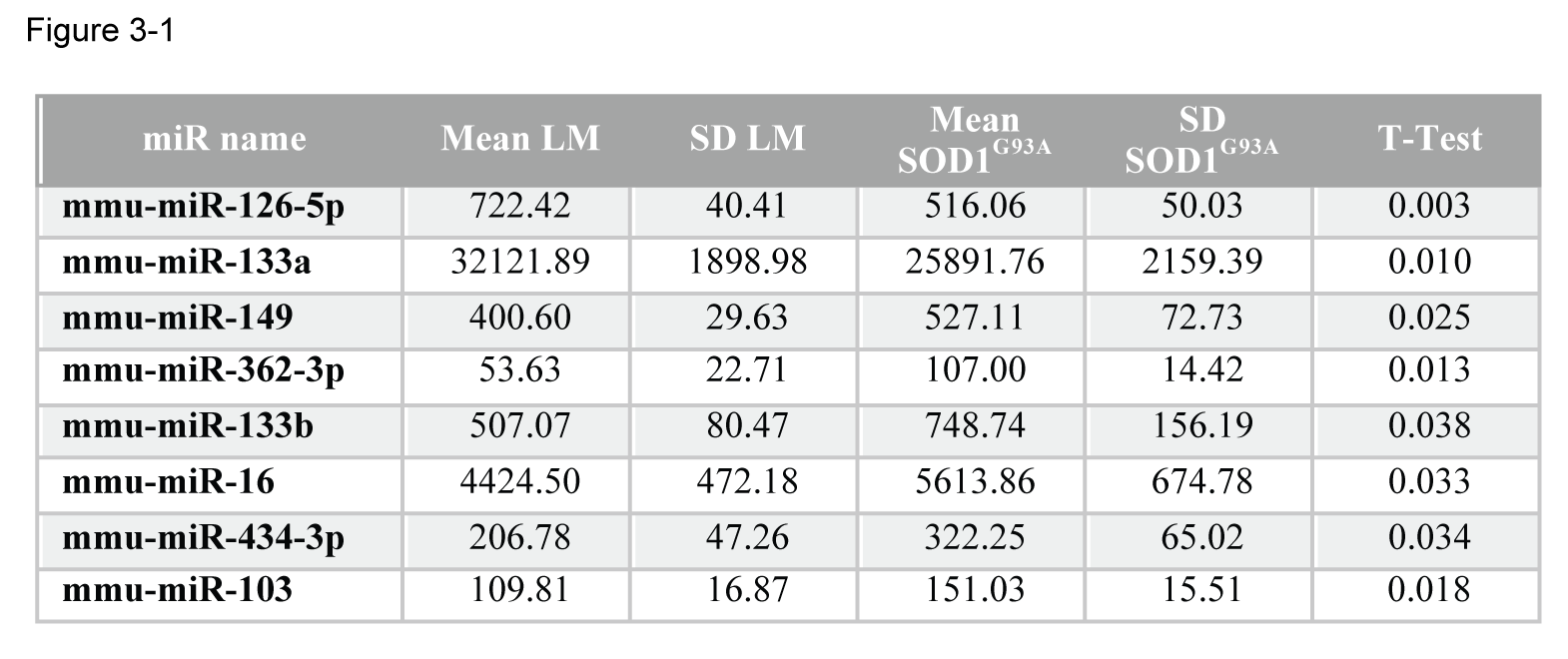

Supplement: Figure 3-1 [file zns999180838so9.tif]

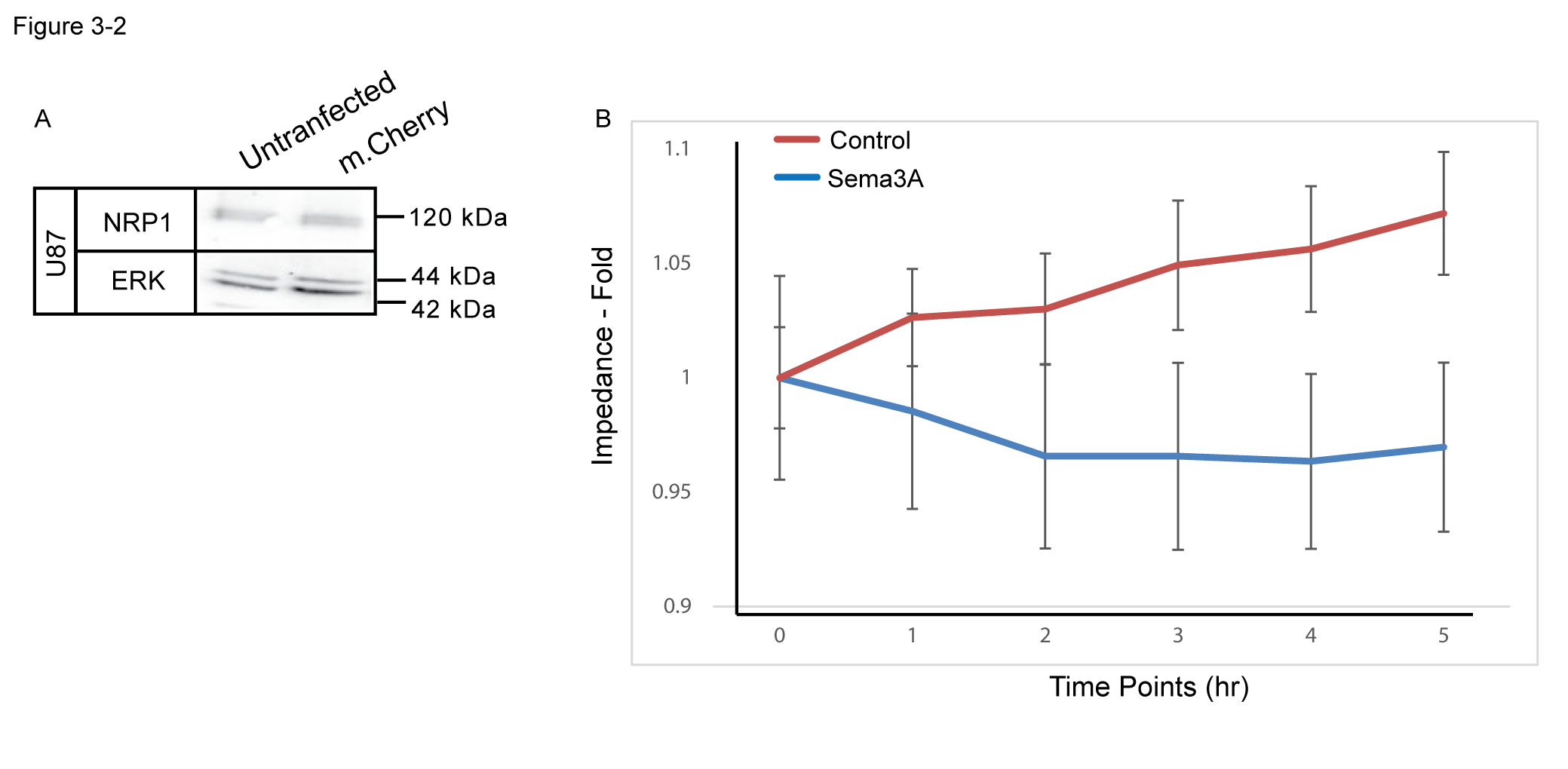

Supplement: Figure 3-2 [file zns999180838so10.tif]

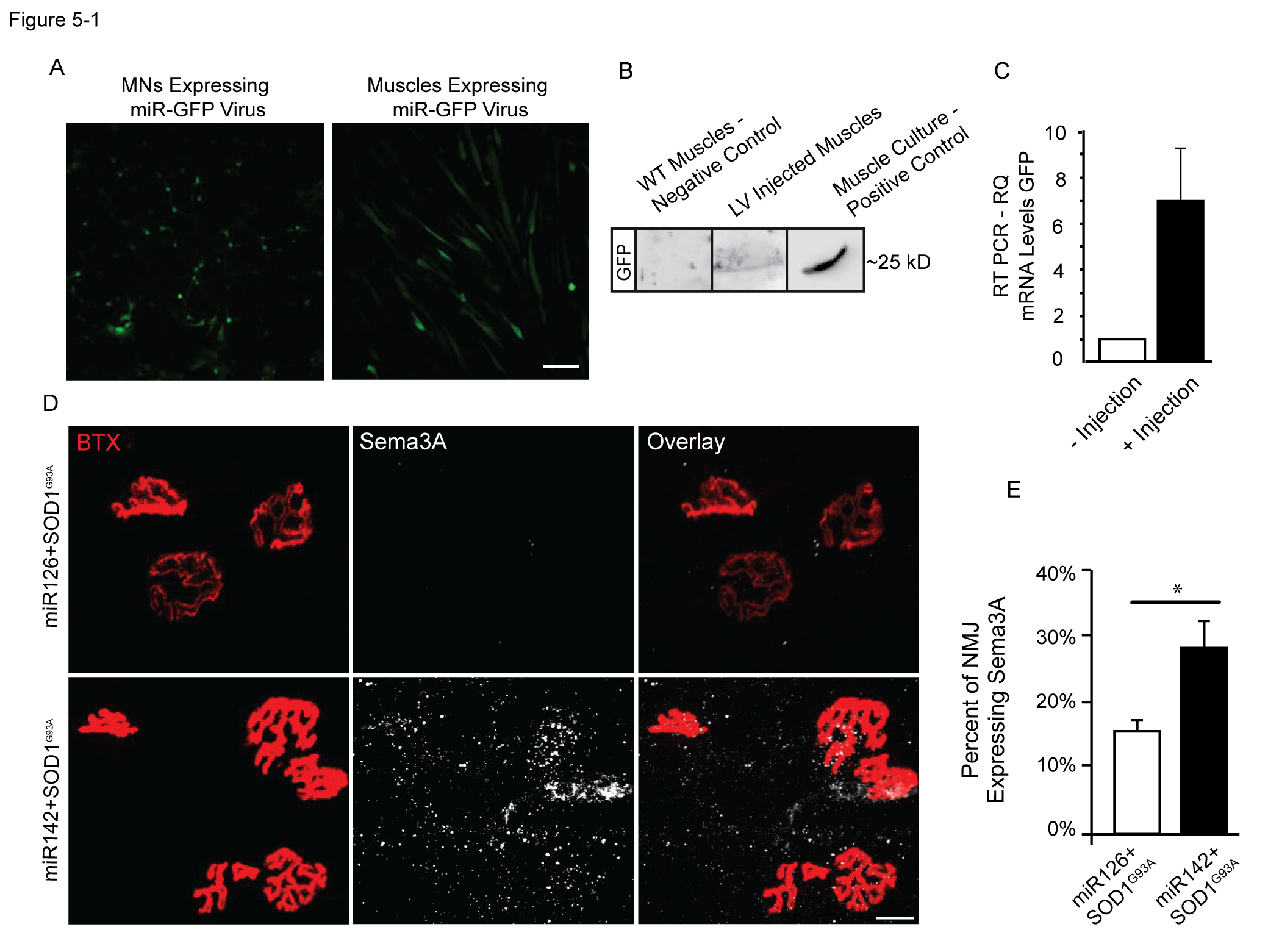

Supplement: Figure 5-1 [file zns999180838so11.tif]
